# ExoS/ChvI Two-Component Signal-Transduction System Activated in the Absence of Bacterial Phosphatidylcholine

**DOI:** 10.3389/fpls.2021.678976

**Published:** 2021-07-23

**Authors:** Otto Geiger, Christian Sohlenkamp, Diana Vera-Cruz, Daniela B. Medeot, Lourdes Martínez-Aguilar, Diana X. Sahonero-Canavesi, Stefan Weidner, Alfred Pühler, Isabel M. López-Lara

**Affiliations:** ^1^Centro de Ciencias Genómicas, Universidad Nacional Autónoma de México, Cuernavaca, Mexico; ^2^Institut für Genomforschung und Systembiologie, Centrum für Biotechnologie (CeBiTec), Universität Bielefeld, Bielefeld, Germany

**Keywords:** *Sinorhizobium meliloti*, membrane lipid, symbiosis, phosphatidylethanolamine, succinoglycan, motility

## Abstract

*Sinorhizobium meliloti* contains the negatively charged phosphatidylglycerol and cardiolipin as well as the zwitterionic phosphatidylethanolamine (PE) and phosphatidylcholine (PC) as major membrane phospholipids. In previous studies we had isolated *S. meliloti* mutants that lack PE or PC. Although mutants deficient in PE are able to form nitrogen-fixing nodules on alfalfa host plants, mutants lacking PC cannot sustain development of any nodules on host roots. Transcript profiles of mutants unable to form PE or PC are distinct; they differ from each other and they are different from the wild type profile. For example, a PC-deficient mutant of *S. meliloti* shows an increase of transcripts that encode enzymes required for succinoglycan biosynthesis and a decrease of transcripts required for flagellum formation. Indeed, a PC-deficient mutant is unable to swim and overproduces succinoglycan. Some suppressor mutants, that regain swimming and form normal levels of succinoglycan, are altered in the ExoS sensor. Our findings suggest that the lack of PC in the sinorhizobial membrane activates the ExoS/ChvI two-component regulatory system. ExoS/ChvI constitute a molecular switch in *S. meliloti* for changing from a free-living to a symbiotic life style. The periplasmic repressor protein ExoR controls ExoS/ChvI function and it is thought that proteolytic ExoR degradation would relieve repression of ExoS/ChvI thereby switching on this system. However, as ExoR levels are similar in wild type, PC-deficient mutant and suppressor mutants, we propose that lack of PC in the bacterial membrane provokes directly a conformational change of the ExoS sensor and thereby activation of the ExoS/ChvI two-component system.

## Introduction

For the establishment of a nitrogen-fixing root nodule symbiosis between legume plants and rhizobial bacteria, a precise temporary sequence of mutual signaling and recognition events is required in order to guarantee a successful outcome for this symbiosis (Gibson et al., 2008). The early events in nodulation are relatively well understood. Plant flavonoids induce nodulation (*nod*) genes most of which are involved in the production of lipochitin oligosaccharide signal molecules (nodulation factors). Nodulation factors are able to induce nodule primordia on respective host plants and thereby trigger the developmental process resulting in nodule formation. Rhizobial exopolysaccharides (EPS), K-like capsular polysaccharides, and cyclic glucans are important secreted compounds required for the infection process by rhizobia as they modulate the host defense response during bacterial invasion of the plant. Subsequently, rhizobial lipopolysaccharides are required for intracellular colonization and for establishing a chronic host infection (Gibson et al., 2008). However, many rhizobial factors, required especially for the late stages of symbiotic development, may still be unrecognized.

In the specific case of *S. meliloti* 1021, which can form a symbiosis with *Medicago sativa* (alfalfa), the synthesis of exopolysaccharide I (EPSI; succinoglycan) or exopolysaccharide II (EPSII; galactoglucan) is required for bacterial advancement in the infection thread and for invasion of the nodule (Cheng and Walker, 1998b). A number of regulatory systems control the formation of EPSI and EPSII (Janczarek, 2011), among them a two-component signal-transduction system that consists of the integral membrane histidine kinase ExoS and the associated cytoplasmic response regulator ChvI (Cheng and Walker, 1998a). The enzymatic activities of the ExoS/ChvI system are controlled by the periplasmic regulator protein ExoR, through direct interaction with ExoS (Wells et al., 2007). Three distinct forms of ExoR are known, the precursor version of ExoR, ExoR_p_, the mature form of ExoR, ExoR_m_, and a shorter, inactive 20 kD form of ExoR, ExoR_c__20_ (Lu et al., 2012). During synthesis of ExoR_p_, the N-terminus is recognized by the type II secretion system, followed by cleavage of the N-terminal signal sequence and transport of the mature ExoR_*m*_ to the periplasm (Wiech et al., 2015). The model for the ExoR-ExoS/ChvI signaling pathway suggests that the ExoS/ChvI system is turned off when the periplasmic domain of ExoS forms a protein complex with the mature periplasmic form ExoR_m_. In the ExoR_m_-ExoS complex, ExoS acts as a phosphatase and keeps ChvI dephosphorylated and inactive. Upon disruption of the ExoR_m_-ExoS interaction, for example, through the proteolytic degradation of ExoR_m_ to ExoR_c__20_, the ExoS/ChvI system becomes derepressed. Signaling by the derepressed ExoS/ChvI system involves autophosphorylation of the transmembrane histidine kinase sensor ExoS, transphosphorylation to the transcriptional regulator ChvI and activation or inhibition of *chvI* box-preceded genes by the phosphorylated ChvI regulator, resulting in upregulation of succinoglycan biosynthesis, repression of flagellum biosynthesis, and altered expression of more than 100 genes, allowing the cells to switch from a free-living to a host-invading form (Yao et al., 2004; Wells et al., 2007; Lu et al., 2012). An extensive list of target genes regulated by ChvI and a consensus sequence for ChvI binding has been identified (Chen et al., 2009; Ratib et al., 2018). The two-component system ExoS/ChvI has not only important roles in succinoglycan production and motility, but also in biofilm formation, nutrient utilization and growth of free-living bacteria (Chen et al., 2009). Mutations in *exoR* or *exoS* that permanently switch on signaling through the ExoS/ChvI system demonstrate reduced efficiency of root hair colonization and a reduction in symbiotic performance, overproduction of succinoglycan, and suppression of flagellum synthesis (Yao et al., 2004). Notably, whereas the *exoS* mutant reported by Yao et al. (2004) is constitutively activated, an *exoS* null mutant has a phenotype similar to a *chvI* mutant, cannot establish an effective symbiosis with *M. sativa* either, and exhibits a pleiotropic phenotype (Bélanger et al., 2009). The mutant deleted in *chvI* failed to grow on complex medium, exhibited lower tolerance to acidic conditions, did not produce EPS or smooth LPS, was hypermotile and symbiotically defective (Wang et al., 2010).

**FIGURE 1:**
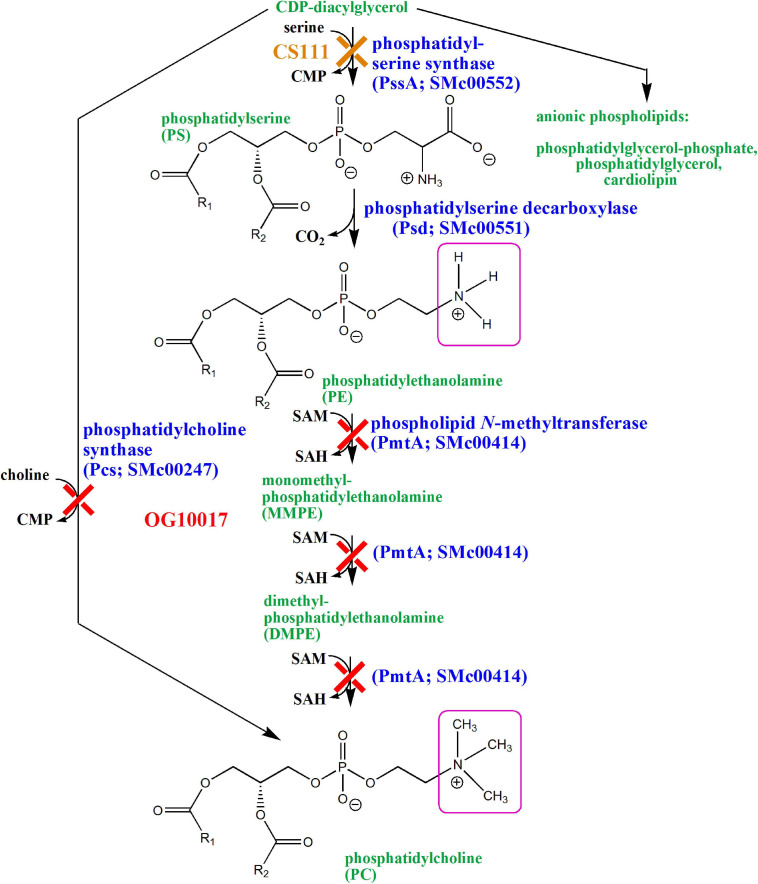
Biosynthesis of phospholipids in *Sinorhizobium meliloti* wild type and in mutants deficient in PE (CS111) or PC (OG10017) formation. Cytidine monophosphate (CMP), cytidine diphosphate (CDP), inorganic phosphate (P_i_), *S*-adenosylmethionine (SAM), *S*-adenosylhomocysteine (SAH). Structural differences between PE and PC are highlighted by pink squares.

The Gram-negative model bacterium *Escherichia coli* has phosphatidylglycerol (PG), cardiolipin (CL), and phosphatidylethanolamine (PE) as major membrane phospholipids. Although phosphatidylcholine (PC) is ubiquitous in eukaryotes, only about 15% of the bacteria are able to synthesize PC for their membranes (Sohlenkamp et al., 2003; Geiger et al., 2013). In *S. meliloti*, both zwitterionic lipids (PE and PC) are major components of the membrane. The only difference between the two types of molecules is that the primary amino group of PE is substituted with three additional methyl groups in the case of PC (Figure 1). In *S. meliloti* and most other bacteria, CDP-diacylglycerol (CDP-DAG) is the central activated lipid precursor for the diversification of membrane lipids with different phosphoalcohol head groups (Figure 1). For example, condensation of serine with CDP-DAG is catalyzed by phosphatidylserine synthase (PssA) and leads to the formation of phosphatidylserine (PS) and CMP (Sohlenkamp et al., 2004). In a second step, phosphatidylserine decarboxylase (Psd) converts PS into PE and CO_2_ (Vences-Guzmán et al., 2008). In *S. meliloti*, PE can be further converted into PC by phospholipid *N*-methyltransferase (PmtA) by triple methylation using *S*-adenosylmethionine as methyl donor and forming *S*-adenosylhomocysteine (de Rudder et al., 2000). *S. meliloti* and many PC-containing bacteria have a second PE-independent pathway for PC formation, the phosphatidylcholine synthase (Pcs) pathway (de Rudder et al., 1999; Sohlenkamp et al., 2000). Pcs catalyzes the condensation of choline with CDP-DAG and forms PC and CMP in one step. In a previous study, we had constructed a double mutant (OG10017) that was deficient in PmtA and Pcs and therefore was unable to form PC (de Rudder et al., 2000; Figure 1). In another study, a mutant (CS111) was constructed that was deficient in PssA and therefore could not form PE (Sohlenkamp et al., 2004). However, CS111 could form PC via the Pcs pathway when cultivated in choline-containing growth media such as complex media containing tryptone and yeast extract (Figure 1). Although the PE-deficient mutant CS111 forms less nodules on legume host plants than the wild type, the mutant was symbiotically effective (Vences-Guzmán et al., 2008). PC occurs in a subset of bacteria many of which are known to interact as pathogens or symbionts with eukaryotic hosts and there has been speculation that bacterial PC might be a requirement for this interaction (de Rudder et al., 1997; Sohlenkamp et al., 2003).

Here we report that a PC-deficient mutant of *S. meliloti* is unable to form a nitrogen-fixing symbiosis with its host plant alfalfa. We show that transcript profiles of mutants deficient in PE or PC differ from each other and are different from the profile of the wild type. Finally, we suggest that the lack of PC in *S. meliloti* activates the ExoR/ExoS/ChvI three-component system which might explain a symbiosis-deficient phenotype of a PC-deficient mutant.

## Results

### PC-Deficient Mutant of *S. meliloti* Cannot Form Nitrogen-Fixing Root Nodules on Alfalfa

**FIGURE 2:**
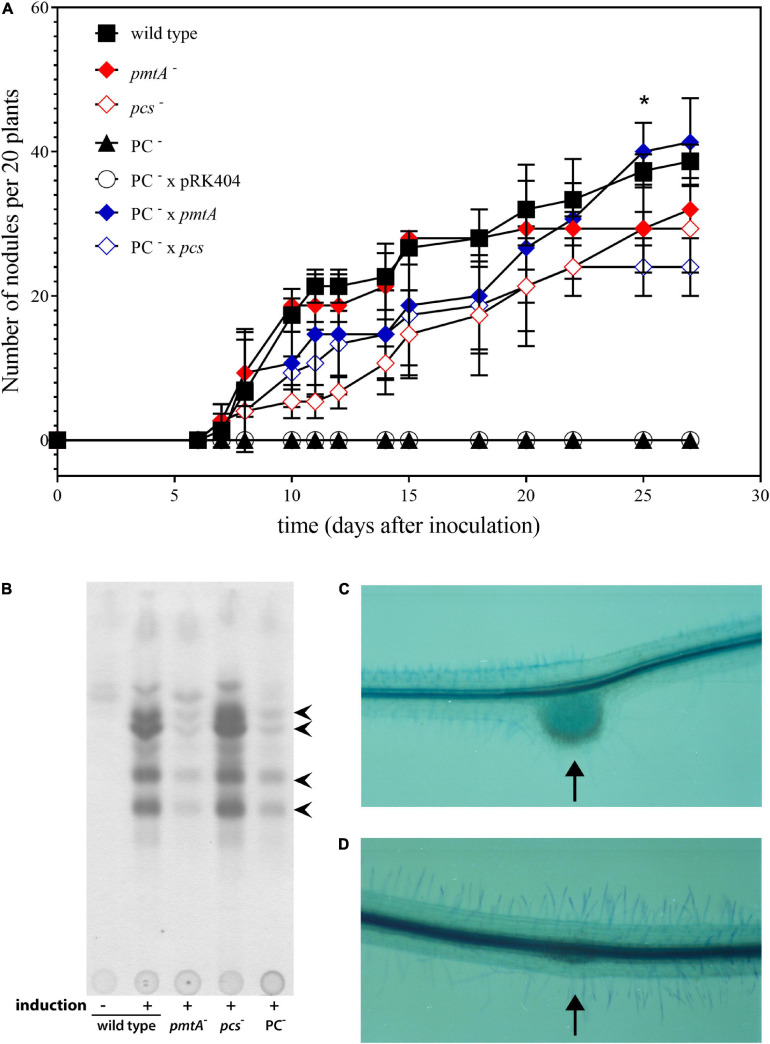
Symbiotic performance of *Sinorhizbium meliloti* strains affected in PC biosynthesis. **(A)** Nodulation kinetics of *S. meliloti* wild type and mutant strains on alfalfa plants. Results for *S. meliloti* wild type strain 1021 (◼), *pmtA*-deficient mutant KDR516 (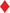), *pcs*-deficient mutant KDR568 (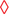), PC-deficient mutant OG10017 (▲),PC-deficient mutant OG10017 harboring the empty plasmid pRK404 (○),PC-deficient mutant OG10017 complemented with *pmtA*-expressing plasmid pTB2042 (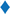), and PC-deficient mutant OG10017 complemented with *pcs*-expressing plasmid pTB2532 (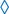). The symbols of ▲ and ○ overlap. Values represent means of three independent experiments and standard deviation is shown. Statistical analysis was performed by a two-way ANOVA with Tukey’s multiple comparisons test as described in Materials and Methods. As an example, comparisons at 25 d after inoculation reveal that nodule numbers of all PC-deficient strains (PC^–^, PC^–^ x pRK404) are significantly different (*p < 0.05) from PC-containing strains (wild type, *pmtA*^–^, *pcs*^–^, PC^–^ x *pmtA*), except *pcs*^–^ versus PC^–^ or *pcs*^–^ versus PC^–^ x pRK404 (*p* < 0.0557). **(B)** PC-deficient *S. meliloti* mutant can form nodulation (Nod) factors. Thin-layer chromatographic analysis of Nod factor formation when *S. meliloti* wild type 1021 was not induced (lane 1) or induced with the flavonoid naringenin (lane 2), or when *pmtA*-deficient mutant KDR516 (lane 3), *pcs*-deficient mutant KDR568 (lane 4), or PC-deficient mutant OG10017 (lane 5) were induced with naringenin. All strains carried plasmid pMP280 for an increased production of Nod factors (Spaink et al., 1987). Arrowheads mark different Nod factors formed by *S. meliloti*. **(C,D)** PC-deficient *S. meliloti* mutant triggers initiation of nodule meristems on alfalfa roots. Arrows indicate a nodule induced by *S. meliloti* wild type (C), or a typical nodule meristem induced by the PC-deficient mutant OG10017 (D), respectively, 10 d after inoculation. For **(C)** and **(D)**, roots were cleared with hypochlorite and stained with methylene blue as described by Truchet and collaborators (Truchet et al., 1989).

In order to study whether bacterial PC was required for the symbiotic interaction of *S. meliloti* with its alfalfa (*M. sativa*) host plant, aseptically grown alfalfa seedlings were inoculated with *S. meliloti* wild type, the *pmtA*-deficient mutant KDR516, the *pcs*-deficient mutant KDR568, the PC-deficient mutant OG10017, OG10017 expressing *pmtA* from plasmid pTB2042, OG10017 expressing *pcs* from plasmid pTB2532, or OG10017 harboring the empty broad host range plasmid pRK404. Of all the strains tested, only the PC-deficient mutant OG10017, which lacks both pathways for PC formation, and OG10017 harboring the empty broad host range plasmid pRK404 were unable to form any nodules on alfalfa host plants (Figure 2A). In contrast, strains with the ability to form PC (wild type, KDR516, KDR568, OG10017 expressing *pmtA*, or OG10017 expressing *pcs*) were able to form pink, nitrogen-fixing root nodules on alfalfa (Figure 2A). A lack of host plant nodulation is often due to a deficient formation and secretion of lipochitin oligosaccharide nodulation factors by the rhizobial microsymbiont (Gibson et al., 2008). However, when analyzing spent culture media we found that *S. meliloti* wild type, the *pmtA*-deficient mutant KDR516, the *pcs*-deficient mutant KDR568, and the PC-deficient mutant OG10017 were able to form several distinct nodulation factors in the typical flavonoid-dependent way (Figure 2B), that were previously shown to constitute a family of structurally related nodulation factors (Schultze et al., 1992). The fact that KDR516 (*pmtA*^–^) and OG10017 (PC^–^) seem to produce less nodulation factors (Figure 2B), is probably due to the reduced growth of both strains on the minimal medium employed. Microscopic analysis of plantlets inoculated either with the wild type or the PC-deficient mutant OG10017, shows that in the case of wild type a typical nodule structure is formed (Figure 2C), whereas in the case of the PC-deficient mutant only an initiating meristem (Figure 2D), that is distinct from side root meristems, is formed. Initiating meristems on alfalfa roots are another indication that the PC-deficient mutant OG10017 can form and secrete nodulation factors in an adequate way.

### Growth Analysis of *S. meliloti* Wild Type and Phospholipid-Deficient Mutants

**FIGURE 3:**
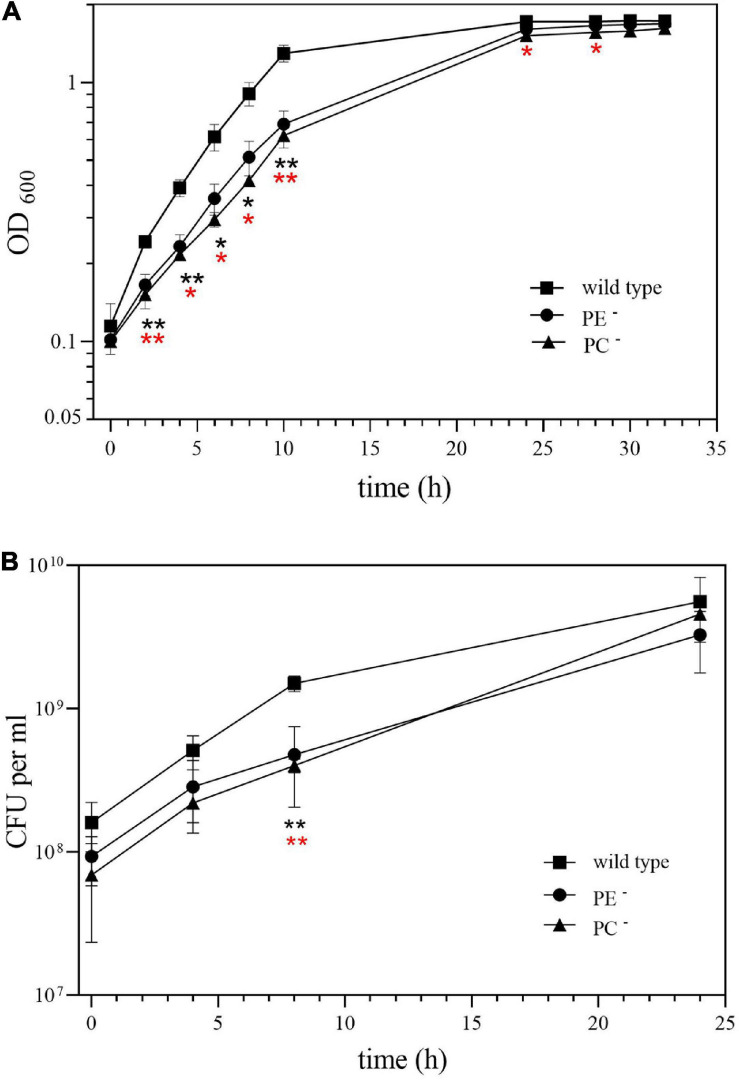
Growth of *Sinorhizobium meliloti* wild type (◼), PE-deficient mutant CS111 (⚫), and PC-deficient mutant OG10017 (▲) on LB/MC+ medium. Growth was recorded by measuring **(A)** the optical density of cultures at 600 nm (OD_600_) or by determining **(B)** the concentration of viable cells as colony-forming units (CFU) per ml. Values represent means of three independent experiments and standard deviation is shown. Statistical analysis was performed by a two-way ANOVA with Tukey’s multiple comparisons test as described in Materials and Methods. Comparisons of the PE-deficient (black asterisks) or the PC-deficient (red asterisks) mutant to wild type are indicated. Statistical significance is shown (*p < 0.05; **p < 0.01).

Our previous results indicated that the PC-deficient mutant OG10017 grew poorly in low osmolarity media such as TY (de Rudder et al., 2000) whereas the PE-deficient mutant CS111 was unable to grow on all the minimal media we tested (Sohlenkamp et al., 2004). In order to get hints why the PC-deficient mutant was symbiotically impaired, we decided to analyze transcript profiles of *S. meliloti* wild type and mutants CS111 (*pssA*^–^; PE^–^) and OG10017 (*pmtA*^–^/*pcs*^–^; PC^–^). Bacterial strains were cultivated on LB/MC+ medium in which they showed similar rates during the exponential phase of growth (Figure 3) following the increase of OD_600_ (Figure 3A) or of colony-forming units (CFU) (Figure 3B) over time. In LB/MC+ medium, the PE-deficient mutant CS111 and the PC-deficient mutant OG10017 showed only slightly reduced growth rates when compared to the wild type and when cultures reached an OD_600_ of 0.8, RNA was isolated and transcriptional profiles established as described in Materials and Methods and in more detail by Hellweg and collaborators (Hellweg et al., 2009). Lipid composition of all three strains after growth on LB/MC+ medium was determined and at those conditions PE is absent in CS111 (*pssA*^–^) and PC is absent in OG10017 (*pmtA^–^/pcs^–^*) (Table 1).

**TABLE 1:** Membrane lipid composition of *Sinorhizobium meliloti* wild type 1021, phosphatidylethanolamine-deficient (*pssA*^–^) mutant CS111, phosphatidylcholine- deficient (*pmtA^–^/pcs^–^*) mutant OG10017 after *in vivo* labeling for 24 h during growth on complex LB/MC+ medium.

strain	wild type	*pssA*^–^	*pmtA^–^/pcs^–^*
PC	75.2 ± 7.6	44.9 ± 3.8	n.d.
PE + (MMPE)	10.2 ± 4.8	n.d.	80.9 ± 5.6
CL	1.4 ± 0.3	16.2 ± 2.7	3.6 ± 2.7
PG	7.1 ± 3.2	33.6 ± 4.8	12.0 ± 2.4
OL	4.1 ± 1.0	1.7 ± 0.8	2.7 ± 0.2
SL	1.2 ± 0.7	3.5 ± 2.1	2.6 ± 1.4
DMPE	0.9 ± 0.5	n.d.	0.5 ± 0.1

*n.d., Not detected; PC, phosphatidylcholine; PE, phosphatidylethanolamine; MMPE, monomethyl-phosphatidylethanolamine; CL, cardiolipin; PG, phosphatidylglycerol; OL, ornithine-containing lipid; SL, sulfolipid; DMPE, dimethyl-phosphatidylethanolamine. Numbers are the mean of three independent experiments ± standard deviation.*

### Transcriptional and Physiological Differences Between Wild Type and PE-Deficient Mutant Cells

**FIGURE 4:**
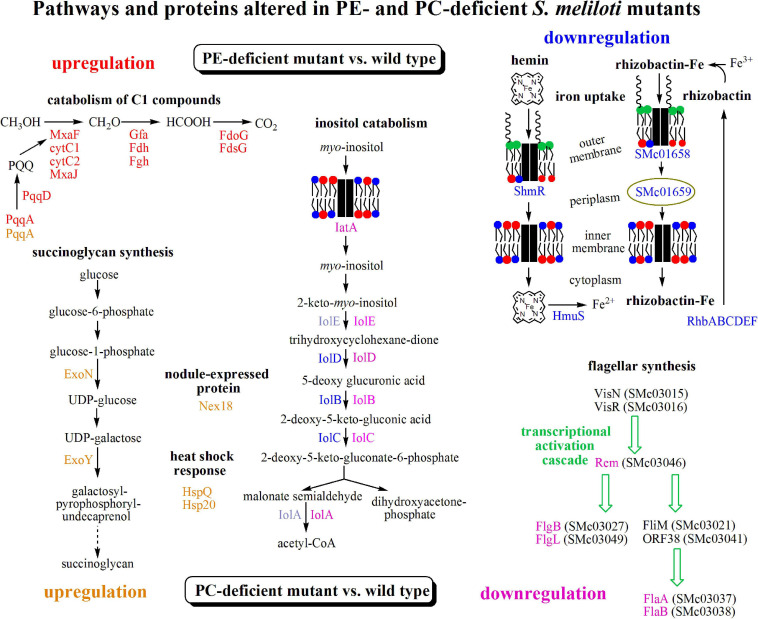
Transcriptomic results suggest differently altered pathways in PE- and PC-deficient mutants of *S. meliloti*. Comparison of transcript profiles of phospholipid-deficient mutants with *S. meliloti* wild type suggests that a pathway for C1 catabolism is upregulated (red) and pathways for inositol catabolism and iron uptake are downregulated (blue) in the PE-deficient mutant CS111 and that the pathway for succinoglycan biosynthesis and heat shock response are upregulated (orange) and pathways for inositol catabolism and flagellar synthesis are downregulated (pink) in the PC-deficient mutant OG10017. Proteins from altered transcripts are highlighted with the respective colors. For details see text, Tables 2, 3, Supplementary Tables 1, 2.

Several differences were observed when the expression profiles of the PE-deficient mutant CS111 and the wild type were compared. In the PE-deficient mutant, transcripts for 11 genes were increased (*M*-value > 1) and transcripts for 12 genes were reduced (*M*-value < −1) (Table 2 and Supplementary Table 1). In CS111, transcripts for the putative quinoprotein methanol dehydrogenase MxaF (SMb20173), cytochromes C (SMb20172, SMb20174), a methanol oxidation protein (SMb20175), and transcripts (SMb20204, SMb20207) required for the formation of the pyrroloquinoline (PQQ) cofactor of bacterial methanol dehydrogenases are increased which would be in agreement with an increased capability of the PE-deficient mutant CS111 to convert methanol to formaldehyde (Figure 4). Also, transcripts for enzymes of the glutathione (GSH)-dependent formaldehyde oxidation (glutathione-dependent formaldehyde-activating enzyme Gfa SMb20186, glutathione-dependent formaldehyde dehydrogenase Fdh SMb20170, *S*-formyl glutathione hydrolase Fgh SMb20171) (Goenrich et al., 2002) are increased in CS111 suggesting an efficient conversion of formaldehyde to formic acid. In this context it is interesting to note that also transcripts for genes (*fdoG*, *fdsG*) of the two functional formate dehydrogenase systems (Pickering and Oresnik, 2008) are slightly increased in CS111 (*M*-value < 1) (Supplementary Table 1) suggesting an increment in the conversion of formic acid to CO_2_. Therefore, the PE-deficient mutant CS111 shows increased transcripts for proteins that might be involved in the degradation of C1 compounds such as methanol or formaldehyde (Figure 4). Presently, we do not know why transcripts for C1 catabolism are increased in the PE-deficient mutant.

**TABLE 2:** Partial list of *S. meliloti* genes differently expressed in the phosphatidylethanolamine-deficient mutant CS111 (M > 1 or M < −1).

Gene	Description	*M*-value
SMb20186 *(gfa)*	glutathione-dependent formaldehyde-activating enzyme	3.58
SMb20204 *(pqqA)*	putative pyrroloquinoline quinone synthesis protein A (PqqA)	3.17
SMb20173 *(mxaF)*	putative methanol dehydrogenase, large subunit	2.20
SMb20171 *(fgh)*	putative *S*-formylglutathione hydrolase	1.45
SMb20325 *(thuE)*	probable trehalose/maltose-binding protein	1.43
SMc02156	conserved hypothetical protein	1.39
SMb20174 *(cytC1)*	putative cytochrome C	1.38
SMc02689	probable aldehyde dehydrogenase	1.33
SMb20172 *(cytC2)*	putative cytochrome C alcohol dehydrogenase subunit	1.24
SMb20175 *(mxaJ)*	putative methanol oxidation protein	1.22
SMa1450	probable thiolase	1.02
SMc01659	putative ATP transporter periplasmic component of high affinity iron transport system	–1.02
SMc00432 *(iolB)*	putative *myo*-inositol catabolism protein	–1.06
SMc01513 (*hmuS*)	putative hemin transport protein	–1.08
SMa2410 (*rhbF*)	rhizobactin siderophore biosynthesis protein RhsF	–1.09
SMc01514	conserved hypothetical protein	–1.11
SMc01163 (*iolY*)	putative bacterial glucose/fructose oxidoreductase	–1.16
SMc01165 (*iolC*)	putative sugar kinase	–1.19
SMc01166 (*iolD*)	putative malonic semialdehyde oxidative decarboxylase	–1.19
SMb20713 *(iatA)*	putative *myo*-inositol transporter, ATP-binding protein	–1.20
SMa2408 *(rhbE)*	rhizobactin siderophore biosynthesis protein	–1.33
SMb20072	putative rhizopine binding protein	–1.62
SMc02726 *(shmR)*	hemin-binding outer membrane receptor	–1.92

*M-values describe the log_2_ ratio between mutant signal/wild type signal as described in section “Materials and Methods.”*

In contrast, several transcripts that might encode proteins required for efficient iron uptake by the hemin or rhizobactin systems are reduced in the PE-deficient mutant CS111 (Figure 4). For example, transcripts for the TonB-dependent, hemin-binding outer membrane protein receptor ShmR (SMc02726), the putative hemin transport protein HmuS (SMc01513) and a gene transcribed in opposite direction encoding the hypothetical protein SMc01514 are reduced (Table 2) which could be explained by an altered regulation through the HmuP regulatory protein (SMc01747)(Amarelle et al., 2010). Also, transcripts for the rhizobactin siderophore biosynthesis proteins RhbABCDEF (SMa2400, SMa2402, SMa2404, SMa2406, SMa2408, SMa2410) (Table 2 and Supplementary Table 1), a TonB-dependent siderophore receptor SMc01658, and a periplasmic component of a high affinity iron transport system (SMc01659) (Supplementary Table 1) seem to be reduced in the PE-deficient mutant CS111 (Figure 4). We therefore speculated that the PE-deficient mutant might suffer from an iron deficiency.

In previous attempts we were unable to obtain sustained growth for the PE-deficient mutant on defined minimal media (Sohlenkamp et al., 2004). We now used a modified MOPS minimal medium in which Ca^2+^ concentration was increased 4-fold, whereas phosphate concentration was reduced 10-fold, and which permitted sustained growth of the PE-deficient mutant CS111 in subcultivations. In earlier experiments we showed that choline contributes to growth of CS111 (Sohlenkamp et al., 2004) and we now show that the addition of Fe^2+^ further stimulates growth of both, the PE-deficient mutant CS111 and the wild type (Supplementary Figure 1). In modified MOPS minimal medium supplemented with choline and Fe^2+^, the time of duplication for the PE-deficient mutant CS111 is maintained at distinct subcultivations (Supplementary Figure 1).

Also, transcripts in rhizopine or inositol catabolism are reduced (Figure 4), among them transcripts for a periplasmic rhizopine-binding protein (SMb20072), a *scyllo*-inositol oxidase IolY (SMc01163), a *myo*-inositol transporter (SMb20713), and *myo*-inositol catabolism proteins IolBCD (SMc00432, SMc01165, SMc01166), suggesting that regulation involving the IolR repressor (SMc01164) (Kohler et al., 2011) is altered in the PE-deficient mutant CS111. Although some *S. meliloti* wild type strains are able to degrade rhizopine, *S. meliloti* 1021 is not (Rossbach et al., 1995). However, *S. meliloti* 1021 can use *myo*-inositol as carbon source. When cultivating *S. meliloti* 1021 on 1/20 LB/MC+ media, the addition of *myo*-inositol clearly stimulates growth and final cell yield of *S. meliloti* 1021 wild type (Supplementary Figure 2) which is also the case for the PC-deficient mutant OG10017 (Supplementary Figure 2). Surprisingly, for the PE-deficient mutant CS111, after initial growth stimulation by *myo*-insitol, growth suddenly stops (Supplementary Figure 2). Determination of colony-forming units after different times clarifies that CS111 is not killed by the presence of *myo*-inositol (data not shown); CS111 rather seems to enter a dormant state when *myo*-inositol is present. Presently we do not know the reason for the *myo*-inositol-provoked growth arrest in the PE-deficient mutant CS111.

### Transcriptional Differences Between Wild Type and PC-Deficient Mutant Cells

Several differences were observed when the expression profiles of the PC-deficient mutant and the wild type were compared. In the PC-deficient mutant, 21 genes were induced (*M*-value > 1) and 11 genes were repressed (*M*-value < −1) (Table 3). The most strongly increased transcript in the PC-deficient mutant OG10017, when compared with wild type, is SMb21440 encoding for a conserved hypothetical protein. Other strongly induced transcripts encode for a putative membrane-bound lytic transglycosylase (SMc01855), for enzymes (UDP-glucose pyrophosphorylase ExoN, galactosyltransferase ExoY) required for the biosynthesis of succinoglycan (exopolysaccharide I) (Figure 4), for the heat shock protein HspQ (SMc00949) and an Hsp20-like heat shock protein (SMb21295), for the symbiotically induced protein Nex18 (SMa1077), and others (Table 3). It is remarkable that many of the induced transcripts encode for small proteins that possess a transmembrane alpha-helix or proteins that have an N-terminal signal sequence destining them for transport by the type II secretion system across the cytoplasmic membrane. In contrast, transcripts encoding proteins required for flagella formation (SMc03038, SMc03037, SMc03049, SMc03027, SMc03046, SMc03051) or pili (SMc04114) are reduced in the PC-deficient mutant OG10017 and therefore the transcriptional activation cascade for flagellar synthesis is expected to be reduced in its function in this mutant (Figure 4). Similarly as noted for the PE-deficient mutant CS111 (Table 2), in the PC-deficient mutant OG10017, a reduction for transcripts encoding for proteins required for *myo*-inositol catabolism (IolD, IatA, IolA) (Figure 4) is observed (Table 3). The complete set of differentially expressed genes for the PC-deficient mutant OG10017 is available (Supplementary Table 2).

**TABLE 3:** Partial list of *Sinorhizobium meliloti* genes differently expressed in the phosphatidylcholine-deficient mutant OG10017 (M > 1 or M < −1).

Gene	Description	*M*-value
SMb21440	conserved hypothetical protein*	2.52
SMc01855	putative lytic transmembrane transglycosylase*	2.06
SMc02156	conserved hypothetical protein*	2.01
SMa1246	conserved hypothetical protein DUF88	1.70
SMa0994	conserved hypothetical protein	1.65
SMa1077 (*nex18*)	symbiotically induced conserved protein*	1.57
SMc00949 (*hspQ*)	heat shock protein	1.53
SMb20204 *(pqqA)*	putative pyrroloquinoline quinone synthesis protein A (PqqA)	1.52
SMb20960 (*exoN*)	UDP-glucose pyrophosphorylase	1.46
SMc03999	hypothetical protein DUF465	1.39
SMb20902	putative sugar uptake ABC transporter, periplasmic solute binding protein precursor*	1.38
SMc02900	conserved hypothetical protein DUF1137	1.35
SMc02051	conserved hypothetical protein DUF1137	1.32
SMc01465 (*creA*)	putative CreA protein*	1.28
SMc02052	conserved hypothetical protein DUF1137	1.27
SMc01580	conserved hypothetical protein*	1.25
SMa1831 (*ureF*)	putative urease accessory protein*	1.24
SMb21295 *(hsp20)*	putative small heat shock protein, hsp20 family	1.14
SMc02266	conserved hypothetical protein	1.11
SMb20946 *(exoY)*	galactosyltransferase	1.06
SMb21337	carbon monoxide dehydrogenase (acceptor part of multienzyme complex, twin-arginine translocation pathway signal)	1.03
SMc00781 *(iolA)*	putative methylmalonate-semialdehyde dehydrogenase	–1.04
SMc20072	putative rhizopine binding protein*	–1.11
SMb20713 *(iatA)*	putative *myo*-inositol ABC transporter, ATP-binding protein	–1.11
SMc04114 (*pilA1*)	putative pilin subunit protein*	–1.16
SMc01166 (*iolD*)	putative malonic semialdehyde oxidative decarboxylase	–1.20
SMc03051 (*flbT*)	putative flagellin synthesis repressor protein	–1.32
SMc03046 (*rem*)	transcriptional regulator of exponential growth motility	–1.36
SMc03027 *(flgB)*	flagellar basal body rod protein	–1.56
SMc03049 *(flgL)*	putative flagellar hook-associated protein	–1.56
SMc03037 *(flaA)*	flagellin A	–2.51
SMc03038 (*flaB*)	flagellin B	–2.79

*indicates a predicted N-terminal signal sequence destining the respective protein for transport and processing by the type II secretion system. *M*-values describe the log_2_ ratio between mutant signal/wild type signal as described in section “Materials and Methods.” As of March 22, 2020 the record for DUF1137 domain-containing proteins was discontinued at the NCBI database.

### PC-Deficient Mutant Is Impaired for Swimming and Overproduces Succinoglycan

**FIGURE 5:**
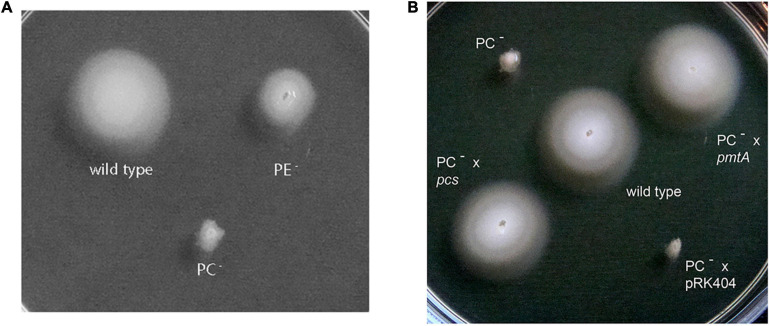
Swimming motility of *Sinorhizobium meliloti* 1021 strains. The assay was performed at 28°C on LB/MC+-containing swim plates (0.3% agar) and analyzed after 2 days. **(A)** *S. meliloti* 1021 (wild type), PC-deficient mutant OG10017, PE-deficient mutant CS111. **(B)** *S. meliloti* 1021 (wild type), PC-deficient mutant OG10017, OG10017 complemented with *pmtA*-expressing plasmid pTB2042, OG10017 complemented with *pcs*-expressing plasmid pTB2532, and OG10017 harboring the empty broad host range plasmid pRK404.

*Sinorhizobium meliloti* wild type, the PE-deficient mutant CS111, and the PC-deficient mutant OG10017 were analyzed for their ability to swim on soft agar plates. Whereas *S. meliloti* wild type and to some lesser extent the PE-deficient mutant exhibited their ability to swim in soft agar (Figure 5A), the PC-deficient mutant did not show any migration away from the initial spot of inoculation (Figure 5). When OG10017 was complemented with the phospholipid *N*-methyltransferase (*pmtA*)-expressing plasmid or with the phosphatidylcholine synthase (*pcs*)-expressing plasmid, swimming was regained which was not the case when an empty broad host range plasmid was employed instead (Figure 5B). As wild type levels of PC can be recuperated in the PC-deficient mutant OG10017 by expressing either *pmtA* or *pcs* (de Rudder et al., 2000), there is a strict correlation between PC formation and the ability to swim in *S. meliloti*.

**FIGURE 6:**
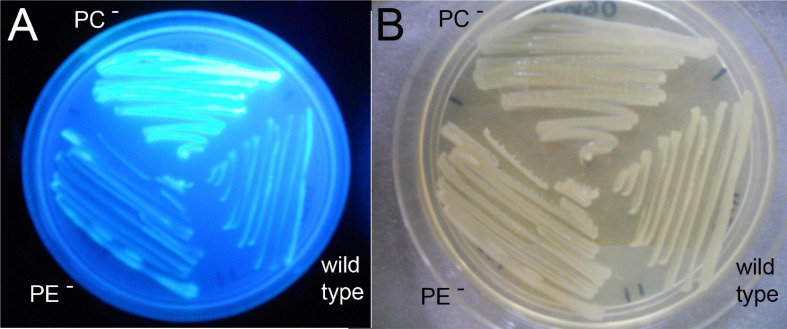
Phosphatidylcholine (PC)-deficient mutant OG10017 shows increased succinoglycan (exopolysaccharide I) formation. *S. meliloti* 1021 (wild type), PC-deficient mutant OG10017, and the PE-deficient mutant CS111 were cultivated for 72 h on LB/MC+-containing agar in the presence of Calcofluor. Upon UV excitation, only the PC-deficient mutant showed much fluorescence indicating overproduction of succinoglycan **(A)**, whereas growth was similar for all three strains **(B)**.

*Sinorhizobium meliloti* wild type, the PE-deficient mutant CS111, and the PC-deficient mutant OG10017 were analyzed for their ability to produce succinoglycan (exopolysaccharide I) on Calcofluor-containing agar plates. Of the three strains only OG10017 provoked a strong fluorescence (Figure 6), indicating that only the PC-deficient mutant OG10017 overproduced succinoglycan while the *S. meliloti* wild type or the PE-deficient mutant CS111 did not. When OG10017 was complemented with the phospholipid *N*-methyltransferase (*pmtA*)-expressing plasmid or with the phosphatidylcholine synthase (*pcs*)-expressing plasmid, succinoglycan was hardly formed as indicated by the lack of fluorescence (Supplementary Figure 3), however, fluorescence was maintained when an empty broad host range plasmid was employed instead (Supplementary Figure 3). Again, as PC formation is recuperated in the PC-deficient mutant OG10017 by expressing either *pmtA* or *pcs*, repression of succinoglycan formation in *S. meliloti* is observed and there is a strict correlation between the presence of PC and reduced formation of succinoglycan in *S. meliloti*.

### PC-Deficient Mutant Is Impaired for Growth at Slightly Acidic pH

**FIGURE 7:**
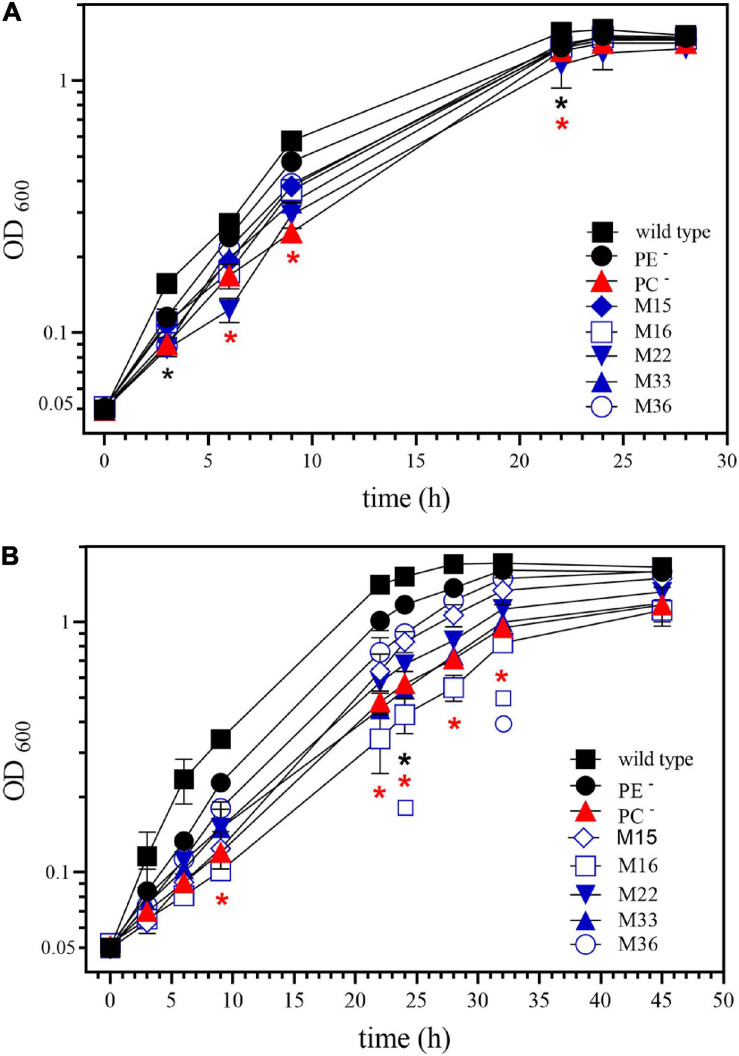
Phosphatidylcholine (PC)-deficient mutant OG10017 is severely impaired in growth on slightly acidic media. Growth of *S. meliloti* wild type strain 1021 (◼), phosphatidylethanolamine (PE)-deficient mutant CS111. (⚫), PC-deficient mutant OG10017 (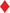), and the correlated suppressor mutants M15 (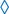), M16 (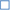), M22 (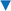), M33 (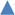), M36 (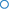) was recorded on LB/MC+ medium containing 20 mM Bis-Tris methane, pH 7.0 **(A)** or on LB/MC+ medium containing 20 mM Bis-Tris methane, pH 5.75 **(B)** by measuring the optical density of cultures at 600 nm (OD_600_). Values represent means of three independent experiments and standard deviation is shown. Statistical analysis was performed by a two-way ANOVA with Tukey’s multiple comparisons test as described in Materials and Methods. Comparisons of the PE-deficient (black asterisks) or the PC-deficient (red asterisks) mutant to wild type are indicated. Statistical significance is shown (*p < 0.05). Also indicated are statistically significant comparisons between the PC-deficient mutant and correlated suppressor mutants M16 (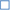) and M36 (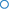).

Interestingly, a shift of *S. meliloti* wild type to slightly acidic pH provokes transcriptomic changes when compared to the transcriptome at neutral pH (Hellweg et al., 2009), that resemble the alterations observed in the PC-deficient mutant when compared with wild type at neutral pH. We analyzed growth of *S. meliloti* wild type, the PE-deficient mutant CS111, and the PC-deficient mutant OG10017. As reported previously (Hellweg et al., 2009), growth of the wild type on buffered LB/MC+ medium at acidic pH 5.75 is only slightly reduced when compared to growth at neutral (pH 7.0) conditions (Figure 7) which holds true for the PE-deficient mutant as well. The PC-deficient mutant OG10017 grows at a similar rate as wild type or PE-deficient mutant at neutral pH (Figure 7A). However, at slightly acidic conditions of pH 5.75, growth of the PC-deficient mutant OG10017 is severely affected (Figure 7B). The lack of PC in mutant OG10017 is in large compensated by PE (Table 1), a lipid with a small head group (Figure 1) causing more negative curvature in each lipid monolayer. PE has a tendency to form the hexagonal II (H_II_) phase and is less prone to form bilayers (Dowhan et al., 2008). Therefore, PC-deficient membranes might be less compact, more permeable for ions and especially for protons.

### Some Mutants Altered in the ExoR/ExoS/ChvI Signal Transduction System Resemble the PC-Deficient Mutant

In trying to understand how PC deficiency is connected on a molecular level to the observed macroscopic and transcriptomic phenotypes, we searched for transcriptome profiles of mutants in the literature that were similarly altered as the one of a PC-deficient mutant. A strain of *S. meliloti* containing a *chvI(D52E)* mutant version in addition to the wild type copy of *chvI* is thought to produce a more active version of ChvI and displays a transcript profile (Chen et al., 2009) that is quite similar to the one we report here for the PC-deficient mutant OG10017 (Table 3 and Supplementary Table 2). From these data one might speculate that, in the absence of PC in sinorhizobial membranes, one or several components of the ExoR/ExoS/ChvI three-component regulatory system are altered resulting in a more active ChvI regulator and the ensuing phenotypes such as increased formation of succinoglycan as well as the absence of flagella and swimming capability.

### Isolation of PC-Deficient Suppressor Mutants That Regain Capacity to Swim and Produce Less Succinoglycan

**FIGURE 8:**
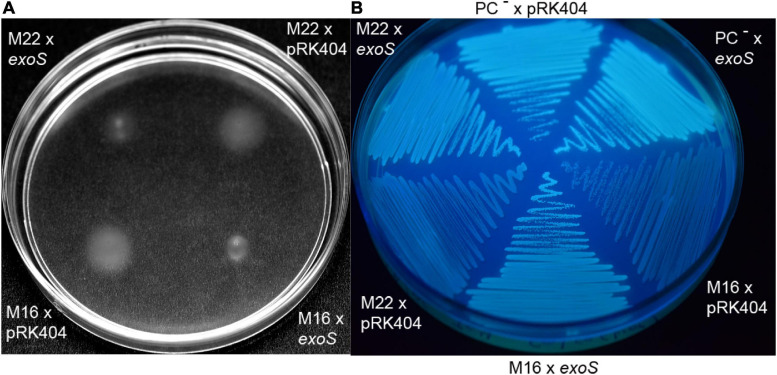
Intact ExoS suppresses swimming and increases exopolysaccharide formation in ExoS-impaired correlated suppressor mutants M16 and M22. **(A)** Swimming of ExoS-impaired correlated suppressor mutants M16 and M22 harboring a plasmid with the wild type version of *exoS* or harboring the empty plasmid pRK404 after 4 d of incubation on LB/MC+-containing swim plates (0.3% agar) in the presence of 4 μg/ml tetracycline. **(B)** Exopolysaccharide production of ExoS-impaired correlated suppressor mutants M16 and M22 harboring a plasmid with the wild type version of *exoS* or harboring the empty plasmid pRK404 after 3 days of incubation.

In order to identify additional elements that might define how PC deficiency causes the loss of swimming and the overproduction of succinoglycan, distinct individual colonies of the PC-deficient mutant OG10017 were used to inoculate each on a swim plate (LB/MC+ medium with 0.3% agar). Though no swimming was observed after 8 d of incubation, possible suppressor mutants for swimming recuperation were sampled with a toothpick from an area that seemed devoid of bacteria and reinoculated on fresh swimming plates. Of about 40 initial isolates, some 30 had regained the capacity to swim after two rounds of selection for swimming. Of these 30 suppressor mutants that regained the capacity to swim, none could form PC, and 5 of these mutants also showed a reduced wild type-like formation of succinoglycan (EPSI). These 5 suppressor mutants (M15, M16, M22, M33, M36) that had regained swimming and formed reduced levels of succinoglycan we named correlated mutants. As mutants altered in components of the ExoR/ExoS/ChvI system usually affect both, swimming and succinoglycan synthesis in a correlated but inverse way, we expected that suppressor mutations in the correlated mutants might map in any of the genes encoding for the three component system ExoR/ExoS/ChvI. The *exoR*, *exoS*, and *chvI* genes as well as their upstream and downstream regions were amplified by PCR from genomic DNA of the 5 respective suppressor mutants and sequenced. Correlated suppressor mutants M15, M33, and M36 displayed *exoR*, *exoS*, and *chvI* genes identical to the wild type. In contrast, M16 had a point mutation T944 to C resulting in an I315T mutation in ExoS and M22 had a mutation G1423 to A resulting in a G475S mutation in ExoS. ExoS contains 595 amino acid residues and the mutation I315T in M16 is located in the HAMP domain, just downstream of the second transmembrane domain/helix of ExoS whereas G475S in M22 is located early in the N box (Cheng and Walker, 1998a) of ExoS. If M16 or M22 were complemented with a wild type version of *exoS*, swimming was suppressed (Figure 8A) and succinoglycan overproduction was recuperated (Figure 8B) which was not the case if an empty broad host range plasmid was present in M16 or M22 (Figure 8). Therefore, the expression of an intact *exoS* gene in M16 or in M22 again reversed the correlated phenotype observed in M16 and M22 and led to similar phenotypes as detected in the PC-deficient mutant OG10017.

### *S. meliloti* Suppressor Mutants M16 and M22 Carry Reduced Function ExoS Mutations

When comparing wild type and PC-deficient mutant in our microarray studies (Table 3 and Supplementary Table 2), we found that transcripts for SMb21440, SMc01855, *exoN*, and *exoY* were increased in the mutant whereas transcripts for *visN*, *rem*, *flgB*, and *flaA* were reduced. Determination of the relative expression of these genes by RT-PCR (Supplementary Figure 4) using specific oligonucleotides (Supplementary Table 3), confirms our previous results that mRNA levels of SMb21440, SMc01855, *exoN*, and *exoY* were increased in PC-deficient mutant OG10017 whereas transcripts for *visN*, *rem*, *flgB*, an d *flaA* were reduced. Expression levels of these target genes in M16 or M22 suppressor mutants were at (SMb21440, SMc01855, and *rem*) or close to (*exoN*, *exoY, visN, flgB*, and *flaA*) wild type levels (Supplementary Figure 4), emphasizing that in the ExoS-altered mutants M16 and M22, well-known ExoS-controlled genes are altered like in ExoS reduced function mutants. Recent studies suggest that ChvI negatively regulates transcription of *rem* directly (Ratib et al., 2018), thereby reducing the transcriptional regulator Rem required for the expression *flgB*, *flaA* and other flagellar genes.

**FIGURE 9:**
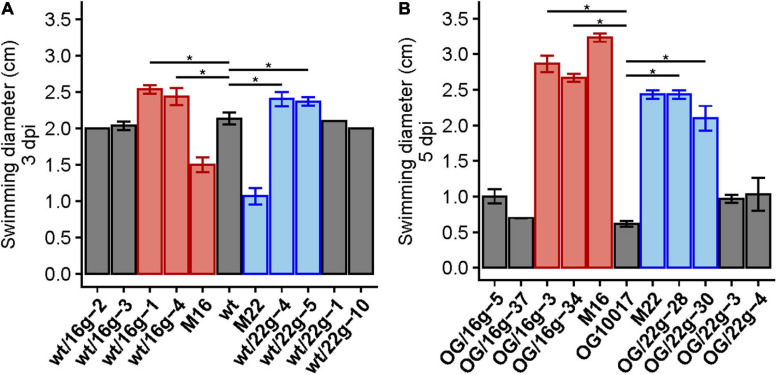
Transduction of M16 and M22 versions of *exoS* increase swimming behavior in wild type and OG10017 backgrounds. Swimming diameters of wild type (wt), correlated suppressor mutants M16 and M22 and some gentamicin-resistant wild type transductants (wt/16g or wt/22g) 3 days post inoculation (3 dpi) **(A)**, as well as swimming behavior of OG10017 (OG), correlated suppressor mutants M16 and M22 and some gentamicin-resistant OG10017 transductants (OG/16g or OG/22g) 5 days post inoculation (5 dpi) **(B)**. Colors indicate the *exoS* version of strains: wt (gray), M16 (red), M22 (blue). The assays were performed at 28°C on LB/MC+-containing swim plates (0.3% agar). Values represent means of three independent experiments and standard deviation is shown. Pairwise comparisons were done using a Wilcox test and denoted by horizontal lines between the compared groups. Statistical significance is shown (*p < 0.05).

In order to show that reduced function mutations of *exoS* in M16 and M22 were linked to an increased swimming phenotype, we introduced a gentamicin resistance-conferring cassette at a distance of 15 kb from the *exoS* gene. Transduction of gentamicin resistance from M16 or M22 to wild type or PC-deficient mutant OG10017 led to the isolation of transductants. Some of these transductants showed increased swimming when compared to their respective parent strains (Figure 9 and Supplementary Figure 5). Sequencing of the *exoS* genes in improved swimming transductants of wild type or OG10017 showed that they carried the *exoS* mutant alleles of M16 or M22, respectively (Figure 9). It is especially remarkable that the *exoS* mutant alleles of M16 or M22 in a PC-replete wild type background display even larger swimming diameter than the *S. meliloti* wild type (Figure 9A), suggesting that both, the presence of PC as well as the presence of the M16 or M22 mutant alleles contribute to obtain an even more inactive ExoS sensor. These data strongly suggest that the reduced function point mutations detected in M16 or M22 can be cotransduced with the gentamicin resistance-conferring cassette and are the cause for the improved swimming phenotypes, respectively.

Restoration of PC biosynthesis in the PC-deficient mutant OG10017 restored the ability to swim (Figure 5 and Supplementary Figure 6). Also in suppressor mutants M16 and M22 swimming behavior is further increased when PC biosynthesis is restored by the action of PmtA or Pcs, which is not the case when M16 or M22 harbored an empty vector (Supplementary Figure 6). Again these data suggest that both, the presence of PC and the presence of the M16 or M22 mutant alleles contribute to reduce ExoS signaling activity in an additive way.

Expression of distinct *exoS* versions in sinorhizobial strains revealed swimming was suppressed slightly in wild type and strongly in M22 when the M16 version of *exoS* was expressed *in trans* (Supplementary Figure 7). Also, when the M16 version of *exoS* was expressed in wild type or M16, exopolysaccharide production was clearly reduced. In contrast, expression of wild type or the M22 versions of *exoS*, did not cause major alterations in swimming behavior or exopolysaccharide production (Supplementary Figure 7). As ExoS can display three distinct enzymatic activities, i.e., autophosphorylation, phosphorylation of ChvI and phosphatase activity on ChvI, it will require future studies of the individual enzyme-catalyzed steps in order to resolve which of the steps might be affected in the respective mutants.

The point mutations encountered in M16 and M22 are both reduced function *exoS* mutations. It is worth noting that while the *exoS* point mutations in M16 and M22 restored wild type-like swimming behavior or exopolysaccharide production, they did not restore wild type-like growth at acidic conditions (pH 5.75). Whereas M22 grows similarly bad as OG10017, M16 grows even worse (Figure 7B). Other, so far uncharacterized, correlated suppressor mutants grew similarly (M33), slightly better (M15), or much better (M36) than OG10017 at acidic conditions (pH 5.75) (Figure 7B). In the future, whole genome sequencing of the DNA from M15, M33, M36 will identify the mutation(s) responsible for the correlated suppressor phenotypes in those mutants.

### ExoR Levels Are Similar in Wild Type, PC-Deficient Mutant and Suppressor Mutants

**FIGURE 10:**
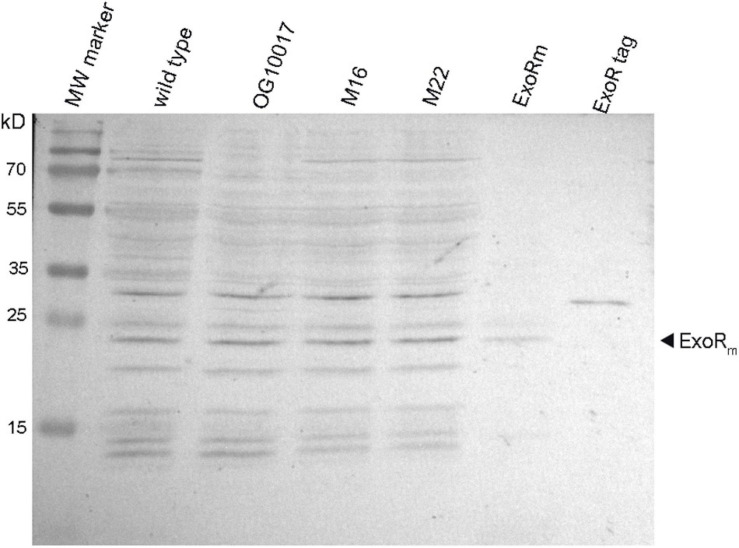
Levels of mature ExoR_m_ are similar in *Sinorhizobium meliloti* wild type and in PC-deficient mutants. Western blot detection of ExoR protein after electrophoretic separation of extracts from *S. meliloti* wild type, OG10017, M16, M22 strains, an extract of *E. coli* BL21(DE3) strain overexpressing ExoR_m_ (ExoRm), and purified ExoR_p_ with an N-terminal His-tag (ExoR tag). For estimation of molecular weights for distinct ExoR versions, a molecular weight (MW) marker (PageRuler Prestained Protein Ladder, Thermo Fisher Scientific) was used.

In PC-deficient mutants of *S. meliloti*, the ExoS/ChvI two-component regulatory system seems to be activated. This regulatory system is usually switched on when the mature and functional form of the periplasmic ExoR regulator protein, ExoR_m_, is proteolytically degraded at acidic conditions (Wu et al., 2012; Wiech et al., 2015). Western blot analyses (Figure 10) indicate that the amounts of ExoR_m_ are similar in *S. meliloti* wild type, the PC-deficient mutant OG 10017 and the correlated suppressor mutants M16 or M22 (Figure 10). We therefore conclude that there is no major alteration of ExoR_m_ levels in *S. meliloti* strains that lack PC. Thus it is unlikely that PC deficiency is mediated through ExoR_m_ and that reduced ExoR_m_ levels in the PC-deficient mutant would activate the ExoS/ChvI two-component regulatory system. We therefore assume that the lack of PC in the cytoplasmic membrane of *S. meliloti* and the concomitant increase of PE directly affect the protein parts of ExoS which are in contact with the membrane, i.e., the two transmembrane helices. We postulate that the orientation of the two ExoS transmembrane helices is altered in a PC-deficient mutant and that these alterations provoke conformational changes that lead to the activation of the ExoS cytoplasmic domain.

## Discussion

### Model for the Regulation of the ExoR/ExoS/ChvI Signal Transduction System

**FIGURE 11:**
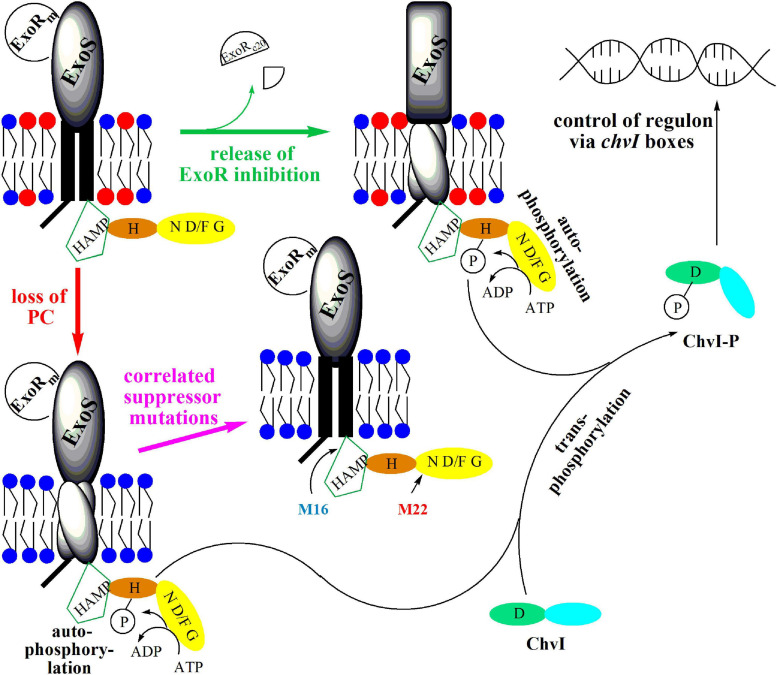
Model for the activation of sensor kinase ExoS by release of ExoR inhibition or by loss of PC from the membrane. In free-living *S. meliloti* bacteria, the two-component regulatory system ExoS/ChvI, remains inactive due to binding of the periplasmic inhibitor protein ExoR_m_ to the ExoS sensor kinase. Upon contact of *S. meliloti* with its legume host, ExoR_m_ is proteolytically degraded, forming ExoR_c__20_, thereby eliminating the inhibition of ExoS and triggering autophosphorylation of ExoS on the conserved H368 residue. Subsequently, the ExoS transphosphorylation activity phosphorylates the conserved D52 residue of the ChvI response regulator. Phosphorylated ChvI-P alters transcription of more than 100 genes, leading for example to the formation of more succinoglycan, less flagellar proteins and to reduced swimming capability. Loss of PC from the membrane probably provokes rearrangement of transmembrane helices 1 and 2 of ExoS, thereby triggering conformational change and activation of the cytoplasmic domain of ExoS. Autophosphosphorylation of ExoS followed by transphosphorylation to ChvI results also in production of more succinoglycan and reduced swimming. Correlated suppressor mutations in M16 (I315T) or M22 (G475S) render the cytoplasmic ExoS domain into a more inactive conformation avoiding autophosphorylation of ExoS, transphosphorylation to ChvI and formation of phosphorylated ChvI-P. Therefore, correlated suppressor mutants M16 and M22 display wild type-like succinoglycan production and swimming behavior. The ExoS protein from *S. meliloti* comprises 595 amino acid residues and contains an N-terminal cytoplasmic domain (residues 1-47), transmembrane helix 1 (residues 48-68), a periplasmic domain (residues 69-278), transmembrane helix 2 (residues 279-299), and a C-terminal cytoplasmic domain (residues 300-595). Within the cytoplasmic domain, a HAMP-domain (residues 301-357) and a histidine kinase domain (residues 365-593) can be distinguished. The HAMP domain is considered crucial for dimerization of sensor kinase monomers and for functionality of the sensor. The histidine kinase domain comprises the conserved H368-containing H box (residues 361-380), as well as the nucleotide binding cleft defined by several conserved amino acids motifs, the N box (residues 473-494), the D/F box (residues 513-540), and the G box (residues 549-567). PC is highlighted by a red circle for its head group, whereas head groups for PE and other membrane lipids are represented by a blue circle.

Our results demonstrate that a PC-deficient mutant of *S. meliloti* is unable to form a nitrogen-fixing root nodule symbiosis with its host plant *M. sativa*, overproduces genes for succinoglycan production and represses genes for flagellum formation. The PC-deficient mutant is indeed unable to swim and overproduces succinoglycan. The transcriptomic phenotype of a PC-deficient mutant resembles that of a strain which is activated in the ExoS/ChvI two-component regulatory system leading to the hypothesis that the absence of PC in *S. meliloti* leads to the activation of ExoS/ChvI. It is generally thought that for the activation of ExoS/ChvI, the inhibition of ExoS by ExoR must be eliminated (Figure 11), probably through proteolytic degradation of the periplasmic ExoR_m_ inhibitor, resulting in activation of the ExoS/ChvI signal transduction system. However, as ExoR_m_levels in the PC-deficient mutant are essentially on the same level as in wild type, we suggest that PC-deficiency is not transmitted to ExoS via ExoR_m_ but in a more direct way. We propose that the lack of PC in the cytoplasmic membrane of *S. meliloti* and the concomitant relative increase of PE cause negative curvature in both monolayers of the cytoplasmic membrane and suggest that this directly affects interactions with the protein parts of ExoS that are in contact with the membrane, i.e., the two transmembrane helices. We postulate that the orientation of the two ExoS transmembrane helices is altered in a PC-deficient mutant and that these alterations provoke conformational changes that lead to the activation of the ExoS cytoplasmic domain, autophosphorylation of ExoS, transphosphorylation to ChvI and alteration of ChvI-controlled gene expression (Figure 11). In the course of this work we have isolated spontaneous suppressor mutants of the PC-deficient mutant OG10017, that had regained swimming and formed reduced levels of succinoglycan. Two of those mutants had distinct point mutations in the *exoS* gene. Mutant M16 had a point mutation T944 to C resulting in an I315T mutation in ExoS and M22 had a mutation G1423 to A resulting in a G475S mutation in ExoS. ExoS contains 595 amino acid residues and the mutation I315T in M16 is located in a domain, present in histidine kinases, adenyl cyclases, methyl-accepting proteins, and phosphatases (HAMP), just downstream of the second transmembrane domain/helix of ExoS. The HAMP domain is critical for dimerization of ExoS monomers and dimerization is a requisite for phosphorylation of the conserved H residue in the H box of one monomer by the phosphorylation domain of the other monomer. In contrast, G475S in M22 is located early in the N box (Cheng and Walker, 1998a) of ExoS. The N box of two-component sensor proteins is part of the nucleotide binding cleft, required for ATP binding and therefore critical for their kinase activity (Figure 11). Transduction of the suppressor point mutations to the PC-deficient mutant OG10017 yielded transductants that regained swimming, suggesting that in these transductants, even in the absence of PC, ExoS signaling is silenced, ChvI remains inactive, and flagellar target genes are expressed. Therefore, the lack of PC in mutant OG10017 must have been transmitted via the ExoS sensor to the genes of the ChvI regulon. Our proposal that membrane composition and properties affect the functioning of two-component system sensors might constitute a more common feature, as also in *E. coli* mutant strains that lack PE, the CpxA/CpxR two-component signal transduction pathway is activated (Mileykovskaya and Dowhan, 1997) and it has been suggested that this activation might occur by envelope stress exerted in the cytoplasmic membrane.

### ExoR/ExoS/ChvI Orthologs and PC Co-occur in the *Rhizobiales*

Orthologs of *S. meliloti* ExoS and ChvI play important roles in other *Rhizobiales* known for their symbiosis with other legumes or for causing plant or animal pathogenesis. The orthologous ChvG/ChvI system of *Rhizobium leguminosarum* is important for successful nodule formation on peas, lentils, and vetch (Vanderlinde and Yost, 2012). The *Agrobacterium tumefaciens* ChvG/ChvI two-component regulatory system controls the expression of acid-inducible genes, bacterial virulence regulation via the VirA/VirG two-component regulatory system, as well as succinoglycan production, and motility (Heckel et al., 2014). The orthologous two-component regulatory system in *Brucella abortus* is BvrS/BvrR; it controls expression of the *Brucella* type IV secretion system and is essential for virulence (Martínez-Nuñez et al., 2010). In the causative agent of cat-scratch disease *Bartonella henselae*, the orthologous BatS/BatR two-component regulatory system controls the adaptive response during infection of human endothelial cells (Quebatte et al., 2010). Whereas ExoS/ChvI orthologs are more widespread in alpha-proteobacteria, orthologs of ExoR seem to be limited to the *Rhizobiales* (Wiech et al., 2015; Heavner et al., 2015) and most of them interact with eukaryotic hosts in a symbiotic or pathogenic way. All the bacteria mentioned above possess PC as a major membrane lipid (Martínez-Morales et al., 2003) and our own analysis (Supplementary Table 4) shows that at least one probable PC biosynthesis pathway exists in all the *Rhizobiales* analyzed, but also in some other alpha-proteobacteria, such as members of the *Acetobacteraceae* (*Gluconobacter*), the *Rhodobacterales* (*Rhodobacter*), or the *Sphingomonadales* (*Sphingomonas, Zymomonas*). Therefore, in all cases where a complete ExoR/ExoS/ChvI (RSI) invasion switch is present, the capability to form PC exists, suggesting that PC is generally required for the proper functioning of the ExoR/ExoS/ChvI system.

### Diverse Roles for PC in Bacteria

The lack of PC in distinct bacteria has phenotypic consequences of varying severity (Geiger et al., 2013). Pcs-deficient mutants of different *Pseudomonas aeruginosa* strains did not produce detectable amounts of PC and behaved like their respective wild types when assayed for many phenotypes, among them motility, biofilm formation, colonization, or virulence (Malek et al., 2012). In the causative agent of Legionnaires’ disease, *Legionella pneumophila*, PC is required for proper functioning of a type IVB secretion system, for cytotoxicity and full virulence, but also for the specific attachment of *L. pneumophila* to macrophages via the platelet-activating receptor (Conover et al., 2008) before entering the host cell. Also, for the animal pathogen *Brucella abortus*, PC is required for full virulence (Comerci et al., 2006; Conde-Alvarez et al., 2006). A PmtA-deficient mutant of *Bradyrhizobium japonicum* showed reduced levels of PC in its membranes, affected nodule development, diminished nitrogen fixation activity and did therefore not form a fully functional symbiosis with its host plant soybean (Minder et al., 2001). Surprisingly, a *B. japonicum* mutant deficient in a toxin-antitoxin-like module was unable to produce any PC and formed less nodules on soybean than the wild type (Miclea et al., 2010). Nevertheless, nitrogen fixation by this PC-deficient strain apparently occurred and therefore there seems to be no absolute requirement of bacterial PC for nitrogen fixation by *B. japonicum* (Miclea et al., 2010). *Agrobacterium tumefaciens* is a tumor-inducing plant pathogen and agrobacterial PC is required for the formation of a type IV secretion system essential for tumorgenesis (Wessel et al., 2006). A proteomic and transcriptomic characterization of a PC-deficient mutant of *A. tumefaciens* indicated that expression of virulence genes in this mutant is dramatically (several hundred-fold) reduced when compared to the wild type, whereas the majority of differentially expressed genes, not related to virulence, was altered only several-fold (Klüsener et al., 2010). Expression of the virulence-related genes is controlled by the two-component regulatory system VirA/VirG and it has been suggested that the VirA/VirG system might not function properly in the PC-deficient mutant of *A. tumefaciens* (Klüsener et al., 2010). We now show that a PC-deficient mutant of *S. meliloti* is unable to form a nitrogen-fixing symbiosis with its host plant alfalfa and we suggest that the lack of PC in *S. meliloti* leads to an activation of the ExoR/ExoS/ChvI three-component. Signaling through ExoR/ExoS/ChvI in *S. meliloti* wild type is normally switched on when the bacterium changes from a free-living life style to a symbiotic one. In contrast, in the PC-deficient mutant this system is already switched on and active when the bacterium gets in contact with the host plant. This premature signaling through ExoS/ChvI might be one reason for the symbiotic deficiency of the PC-deficient mutant.

## Materials and Methods

### Bacterial Strains, Media and Growth Conditions

The bacterial strains and plasmids used and their relevant characteristics are shown in Table 4. *S. meliloti* strains were usually grown at 30°C either in complex LB/MC+ medium, which contained 10 mM of CaCl_2_ instead of 2.5 mM as described previously for LB/MC medium (Glazebrook and Walker, 1991). As PE-deficient mutants require elevated concentrations of bivalent cations for good growth (Sohlenkamp et al., 2004), we used 10 mM CaCl_2_ in culture media in order to obtain similar growth for *S. meliloti* wild type, the PE-deficient mutant CS111, and the PC-deficient mutant OG10017. For cultivation of strains at different pH-values, Bis-tris methane/HCl of different pH-values was added to LB/MC+ medium to a final concentration of 20 mM. Utilization of compounds as carbon or energy sources was evaluated using 1/20 LB/MC+ medium containing reduced concentrations of tryptone (0.5 g/l) and yeast extract (0.25 g/l), normal concentrations of NaCl (5 g/l), MgSO_4_ (2.5 mM) and CaCl_2_ (10 mM), the compound to be evaluated and determining final growth yields (OD_600_). Growth of *S. meliloti* strains on defined media was performed on MOPS minimal medium (Bardin et al., 1996) or, for growth experiments with the PE-deficient mutant CS111, on a modified version of MOPS minimal medium containing 40 mM 4-morpholinepropanesulfonic acid (MOPS), 20 mM KOH, 20 mM NH_4_Cl, 100 mM NaCl, 2 mM MgSO_4_, 5 mM CaCl_2_, 0.3 mg biotin/l, 15 mM succinate, 0.1 mM potassium phosphate buffer, pH 7. *E. coli* strains were grown on Luria-Bertani medium (Miller, 1972) at 37°C. Antibiotics were added to the medium in the following concentrations when required (in μg per ml): 200 for neomycin, 400 for spectinomycin, 40 for gentamicin, 30 for nalidixic acid, 4 for tetracycline for *S. meliloti* and 100 for carbenicillin, 50 for kanamycin, and 20 for tetracycline for *E. coli*.

**TABLE 4:** Bacterial strains and plasmids used in this study.

Strain or plasmid	Relevant characteristics	References
*S. meliloti* 1021our	SU47rf*r*-21	López-Lara et al., 2005
Sm1021our		
derivatives		
CS111	*pssA* gene replaced with gentamicin resistance cassette, cannot form PE	Sohlenkamp et al., 2004
KDR516	*pmtA::kan*, Nm^*R*^	de Rudder et al., 2000
KDR568	*pcs::aadA*, Spc^*R*^	Sohlenkamp et al., 2000
OG10017	*pmtA::kan, pcs::aadA*, cannot form PC	de Rudder et al., 2000
M16	OG10017 derivative carrying a mutated *exoS* (T944C) gene	This work
M22	OG10017 derivative carrying a mutated *exoS* (G1423A) gene	This work
Sm1021g	*S. meliloti* 1021our derivative harboring a gentamicin resistance cassette between SMc02762 and SMc02761 gene	This work
M16g	M16 derivative harboring a gentamicin resistance cassette between SMc02762 and SMc02761 gene	This work
M22g	M22 derivative harboring a gentamicin resistance cassette between SMc02762 and SMc02761 gene	This work
*E. coli*		
DH5α	*recAl* Φ80 *lacZΔM15;* cloning strain	Hanahan, 1983
S17-1	*thi, pro*, recA, *hsdR, hsdM*+, RP4Tc::Mu, Km::Tn7;Tp^*R*^, Sm^*R*^, Spc^*R*^,	Simon et al., 1983
BL21(DE3)	Expression strain	Studier et al., 1990
Plasmids		
pUC19	Cloning vector, Cb^*R*^	Yanisch-Perron et al., 1985
pLysS	Production of lysozyme for repression of T7 polymerase, Cm^*R*^	Studier et al., 1990
pMP280	IncP broad-host range vector, carrying *nodD* promoter and *nodD* gene from *Rhizobium leguminosarum*, Tc^*R*^	Spaink et al., 1987
pMP3510	Broad-host range vector, Tc^*R*^	Spaink et al., 1995
pRK404	Broad-host range vector, Tc^*R*^	Ditta et al., 1985
pTB2042	*pmtA*-containing 2.9 kb *Pst*I/*Pst*I insert in pRK404	de Rudder et al., 2000
pTB2532	*pcs*-containing 4.6 kb *Hin*dIII/*Hin*dIII insert in pRK404	Sohlenkamp et al., 2000
pJMV01	*exoS* gene of *S. meliloti* including 412 b upstream and 29 b downstream, cloned as *Bam*HI fragment in pRK404	This work
pLMA16	mutated *exoS* (T944C) gene of *S. meliloti* including 412 b upstream and 29 b downstream, cloned as *Bam*HI fragment in pRK404	This work
pLMA22	mutated *exoS* (G1423A) gene of *S. meliloti* including 412 b upstream and 29 b downstream, cloned as *Bam*HI fragment in pRK404	This work
pLMA38R	*exoR*_*p*_, cloned as *Nde*I/*Bam*HI fragment in pET16b	This work
pLMA92R	*exoR*_*m*_, cloned as *Nde*I/*Bam*HI fragment in pET9a	This work

*Cb^*R*^, Nm^*R*^, Sm^*R*^, Spc^*R*^, Cm^*R*^ and Tp^*R*^: carbenicillin, neomycin, streptomycin, spectinomycin, chloramphenicol, and trimethoprim resistance, respectively.*

Broad host range plasmids, such as pRK404 derivatives were mobilized into *S. meliloti* strains by diparental mating using the *E. coli* S17-1 donor strain as described by Simon et al. (1983).

### Detection of Lipochitin Oligosaccharide Nodulation Factors by Thin-Layer Chromatography

Cultures of *S. meliloti* harboring plasmid pMP280 were grown on MOPS minimal medium (Bardin et al., 1996) at 30°C on a gyratory shaker in the presence of 2 μg/ml tetracycline. *In vivo* labeling of lipochitin oligosaccharides (LCOs) was carried out in 1 ml cultures at an initial cell density of OD_600_ = 0.06, in the presence of 0.4 μCi of D-[1-^14^C]glucosamine (54 mCi/mmol; Amersham), and in the cases of induction, naringenin (2 μg/ml final concentration) was added. After overnight growth, the LCOs were isolated from the cultures by n-butanol extraction. Samples were concentrated by evaporation and chromatographed on reversed phase C_18_-coated silica plates (Sigma) using acetonitrile/water (1:1; vol/vol) as mobile phase similarly as described (López-Lara et al., 1995). The developed chromatogram was subjected to autoradiography by exposure to a Kodak X-OMAT XK1 film.

### Transcriptional Profiling Using the SM14kOligo Whole Genome Microarray

For microarray hybridization two independent bacterial cultures from each *S. meliloti* strain were grown in LB/MC+ medium to an OD_600_ of 0.8. RNA isolation was performed according to the protocol published by Rüberg et al. (2003) using the RNeasy mini kit (QIAGEN, Hildesheim, Germany). Total RNA (10 μg) was used for the preparation of Cy3 and Cy5 labeled cDNAs. To each SM14kOligo microarray slide (Krol and Becker, 2004) the cDNA of the two respective *S. meliloti* strains were mixed and hybridized. Analysis of microarray images was carried out applying the ImaGen 5.0 software (Biodiscovery Inc., Los Angeles, CA, United States) as described previously (Hellweg et al., 2009). Normalization and significance tests were performed with the EMMA software (Dondrup et al., 2003). *M*-values (log_2_ ratio between both channels), *P*-values (*t*-test) and *A*-values (log_2_ of combined intensity of both channels) were also calculated with EMMA. Detailed protocols and raw data resulting from the microarray experiments have been deposited in the ArrayExpress database with the accession number E-MTAB-3676.

### Reverse Transcription Quantitative Real-Time PCR

Reverse transcription quantitative real-time PCR (RT-qPCR) was essentially performed as described (Nogales et al., 2010). In short, total RNA (1 μg) treated with RNase-free DNase I (Thermo Scientific) was transcribed reversely using Superscript II reverse transcriptase (Thermo Scientific) and random hexamers (Thermo Scientific) as primers. Quantitative real-time PCR was performed on a StepOnePlus (Applied Biosystems). Each 25 μl reaction contained 1 μl of the cDNA, 200 nM of each primer and iQ SyBrGreen Supermix (Thermo Scientific). In order to confirm the absence of contaminating genomic DNA, control PCR reactions of the RNA samples not treated with reverse transcriptase were also performed. By heating at 95°C for 3 min, samples were initially denatured and a 35-cycle amplification and quantification program (95°C for 30 s, 55°C for 45 s, and 72°C for 45 s) followed. The oligonucleotide sequences for qPCR are listed in Supplementary Table 3. The expression levels of selected genes in the mutants were normalized to the expression levels in the wild type strain. The fold change in gene expression was calculated using the comparative critical threshold (ΔΔC_*T*_) method (Schmittgen and Livak, 2008).

### Motility Assays

Swim plates (LB/MC+ medium with 0.3% agar) were point-inoculated with a toothpick and usually incubated for 48 h at 28°C similarly as described (Medeot et al., 2010). Swimming was assessed qualitatively by examining the circular turbid zone formed by the bacterial cells migrating away from the point of inoculation.

### Exopolysaccharide Production

Calcofluor white M2R (fluorescent brightener 28, Sigma) was added to a final concentration of 0.02% in LB/MC+ agar media and production of succinoglycan (exopolysaccharide I) was evaluated based on fluorescence intensity using a hand-held long-wave UV lamp (Leigh et al., 1985).

### DNA Manipulations

Recombinant DNA techniques were performed following standard protocols (Sambrook and Russell, 2001). Commercial sequencing of amplified genes was performed by Eurofins Medigenomix (Martinsried, Germany).

### Cloning and Expression of *exoS* and *exoR* Genes From *S. meliloti*

Using PCR and specific oligonucleotides (AAAGGATCCGGCGTCGGCTATCGCTTCCGCG and AAAGGATCCGGTCCCGTGGACATTGACGAAGG), the genes coding for *exoS* wild type as well as the M16 and M22 mutant versions, including 412 bp upstream and 29 bp downstream, were amplified from the respective genomic DNAs. Suitable restriction sites (underlined) for cloning of the genes were introduced by PCR with the oligonucleotides. After restriction with *Bam*HI, the PCR-amplified DNA fragments were cloned into pUC19 for DNA sequencing. The *Bam*HI-restricted fragments of the wild type *exoS* or the M16 or M22 mutant versions were recloned into a pRK404 vector to obtain pJMV01, pLMA16 or pLMA22, respectively, for expression of the *exoS* wild type gene or the altered M16 or M22 versions in *S. meliloti*.

In order to clone genes coding for ExoR_*m*_ or for a His-tagged version of ExoR_*p*_, PCR and specific oligonucleotides (GGAATTCCATATGTTCGATCCCGGAGCCGGCGT and GGGGATCCTATCAATCGTCGTCGTTCTGC for ExoR_*m*_ or GGAATTCCATATGATGCGCGCGGGTGAATTGAAGTC and CCGGATCCTCAATCGTCGTCGTTCTGCAGATGCA for His-tagged ExoR_*p*_) were employed. Suitable restriction sites (underlined) for cloning of the genes were introduced by PCR with the oligonucleotides. After restriction with *Nde*I/*Bam*HI, the PCR-amplified DNA fragments were cloned into pET9a (for ExoR_*m*_) yielding plasmid pLMA92R or pET16b (for ExoR_*p*_) yielding plasmid pLMA38R and the correct sequences for cloned DNA fragments were corroborated. After transformation of these plasmid constructs to *E. coli* BL21(DE3) x pLysS, transformed strains were cultivated at 29°C and genes were expressed with 0.1 mM IPTG. Cell suspensions of *E. coli* BL21(DE3) x pLysS x pLMA92R were prepared for Western blot analysis, whereas His-tagged ExoR_*p*_ was purified from cell-free extracts of *E. coli* BL21(DE3) x pLysS x pLMA38R by Ni affinity chromatography before being analyzed in Western blots.

### Insertion of a Gentamicin Resistance-Conferring Cassette Into the Genome of *S. meliloti* at a Distance of 15 kb From *exoS*

In order to insert a gentamicin resistance-conferring cassette into the genome of *S. meliloti*, first genomic fragments involving the end of the TRm22 (SMc02762) gene or the end of the *trxA* (SMc02761) gene were amplified by PCR from genomic DNA of *S. meliloti* using specific pair of oligonucleotide primers. Amplification with primers AAATCTAGACGCTGGGTAAGCTGTACGATTG and ACTGGATCCAGACCATTTGGTTTGGGATG yielded a 771 bp fragment, containing part of TRm22, and introduced an *Xba*I and *Bam*HI site, respectively, whereas amplification with primers ACTGGATCCCCGCTATGCATGAATTTTCC and ACTGAATTCACGATCGCCGATACAAGAC produced a 776 bp fragment, containing part of *trxA*, and introducing an *Bam*HI and *Eco*RI site, respectively. After digesting both fragments with the restriction enzymes mentioned, they were cloned into plasmid pK18*mobsacB* (Schäfer et al., 1994). Subsequently, a gentamicin cassette derived from pACΩ-Gm (Schweizer, 1993) was obtained and cloned into the *Bam*HI site of the previous plasmid yielding plasmid pLMA71. The suicide vector pLMA71 was introduced into wild type *S. meliloti* 1021, and double recombinants in which the gentamicin resistance-conferring cassette had been inserted between *trxA* and TRm22 at a distance of about 15 kb from the *exoS* gene were obtained following a procedure described previously (Sohlenkamp et al., 2004). Double recombinants, i.e., Sm1021g, were confirmed by PCR. General transduction employing the phage M12 (Finan et al., 1984) was used to move the gentamicin resistance-conferring cassette from the *S. meliloti* wild type background to M16 and M22. Gentamicin-resistant derivatives of M16 and M22 were isolated and when they resembled M16 or M22 in their swimming behavior, the maintenance of the altered *exoS* genes of M16 or M22, i.e., in M16g or in M22g, respectively, was confirmed by sequencing of the PCR-amplified *exoS* gene. Subsequently, the gentamicin resistance was transduced from the M16g or M22g to the *S. meliloti* wild type or the PC-deficient mutant OG10017 and transductants were isolated. For some of the transductants, the *exoS* gene was PCR-amplified and sequenced and transductants were identified which harbored wild type or M16 versions of *exoS* when M16g was employed as donor or wild type or M22 versions of *exoS* with M22g as donor.

### *In vivo* Labeling of *Sinorhizobium meliloti* With [^14^C]Acetate and Quantitative Analysis of Lipid Extracts

The lipid compositions of *S. meliloti* 1021 wild type and mutant strains were determined following labeling with [1-^14^C]acetate (55 mCi/mmol; Perkin Elmer). Cultures (1 ml) of wild type and mutant strains in LB/MC+ medium were inoculated from precultures grown in the same medium. After addition of 0.5 μCi [^14^C]acetate to each culture, the cultures were incubated for 24 h. The cells were harvested by centrifugation, washed with 500 μl of phosphate-buffered saline (PBS)(Sambrook and Russell, 2001) and resuspended in 100 μl of PBS. The lipids were extracted according to Bligh and Dyer (Bligh and Dyer, 1959) substituting water with PBS. The chloroform phase was used for lipid analysis on thin-layer chromatography (TLC) plates (HPTLC aluminum sheets, silica gel 60, Merck) and after one-dimensional or two-dimensional separation using the solvent systems described (de Rudder et al., 1997), the individual lipids were quantified using a phosphorimager (Storm 820, Molecular Dynamics).

### Preparation of ExoR Antibodies and Their Purification

His-tagged ExoR_*p*_ (ExoR tag) was purified from cell-free extracts of *E. coli* BL21(DE3) x pLysS x pLMA38R by Ni affinity chromatography as described above and 400 μg of the purified protein were injected into a rabbit on four occasions, respectively, at intervals described previously (Cooper and Paterson, 2008). The serum of the rabbit contained antibodies against the ExoR tag protein as determined by testing serial dilutions on immunoblots.

Purification of the ExoR antibody was performed with the ExoR tag protein essentially as described (Scharf et al., 2001). Only during the washing steps of the filter chips, 0.5% BSA and 0.5% Nonidet P40 was used instead of 0.1% BSA and 0.1% Nonidet P40.

### Detection of Different Forms of ExoR by Western Blot Analysis

Cultures of *S. meliloti* strains were grown in LB/MC+ medium to an OD_600_ of 0.8. Aliquots (500 μl) of cultures were quickly harvested for 2 min at 10000 rpm in an Eppendorf centrifuge and resuspended in 50 μl of 50 mM Tris/HCl buffer, pH 7.4 containing 100 mM KCl. An equal volume of 2 × treatment buffer was added to the resuspended cells and the mixture was boiled for 10 min. Protein samples, usually corresponding to 100 μl of the liquid cell cultures, were analyzed by SDS-containing polyacrylamide gel electrophoresis (15%). Separated proteins were transferred to a polyvinylidene difluoride membrane and blots were incubated with ExoR-specific purified antibodies in a 1:300 dilution, as primary antibody and anti-rabbit serum (1:4000 dilution) coupled with alkaline phosphatase as secondary antibody. Blots were developed by treatment with nitro-blue tetrazolium and 5-bromo-4-chloro-3′-indolylphosphate (Sigma).

### Plant Assays

Alfalfa (*Medicago sativa* L.) plants were grown hydroponically on a nitrogen-free medium as described by Olivares and associates (Olivares et al., 1980), which contained about 0.7 mM phosphate. To test the infectivity of the rhizobial strains, 30 individual plants were inoculated with 10^5^ cells. After inoculation the number of nodulated plants and the number of nodules per plant were recorded every three days until no more changes in the total nodule numbers were observed. Plants were incubated in a plant growth chamber at 22°C using a 12-/12-h day/night cycle.

### Statistical Analysis

A two-way ANOVA with Tukey’s multiple comparisons test was conducted on the mean of each OD/CFU/nodule value measured by triplicate to evaluate that observed changes were statistically significant. A *p*-value < 0.05 was considered statistically significant. Data are presented as mean ± standard error of the mean. The analysis was conducted using Graphpad Prism Software version 9.02 (GraphPad Software, San Diego, CA, United States)^1^.

## Data Availability Statement

The datasets presented in this study can be found in the ArrayExpress database (www.ebi.ac.uk/arrayexpress) with the accession number E-MTAB-3676 and as Supplementary Tables S1, S2.

## Author Contributions

OG, CS, SW, AP, and IL-L designed the study. OG, CS, DV-C, DM, LM-A, and IL-L carried out the experiments. OG, CS, DV-C, DM, LM-A, DS-C, SW, AP, and IL-L carried out the data analysis and discussed the results. OG, AP, and IL-L were involved in drafting the manuscript and all authors, except SW who passed away, read and approved the final manuscript.

## Conflict of Interest

The authors declare that the research was conducted in the absence of any commercial or financial relationships that could be construed as a potential conflict of interest.

## Publisher’s Note

All claims expressed in this article are solely those of the authors and do not necessarily represent those of their affiliated organizations, or those of the publisher, the editors and the reviewers. Any product that may be evaluated in this article, or claim that may be made by its manufacturer, is not guaranteed or endorsed by the publisher.

## Footnotes

1 www.graphpad.com
