# Supplementary material for: ExoS/ChvI Two-Component Signal-Transduction System Activated in the Absence of Bacterial Phosphatidylcholine
Source: Front Plant Sci. 2021 Jul 23;12:678976. doi: 10.3389/fpls.2021.678976 (PMC8343143; doi:10.3389/fpls.2021.678976)
Supplement: Supplementary file 1 [file Data_Sheet_1.PDF]

ExoS/ChvI two-component signal-transduction system activated in the absence of bacterial phosphatidylcholine

Supplementary Figures

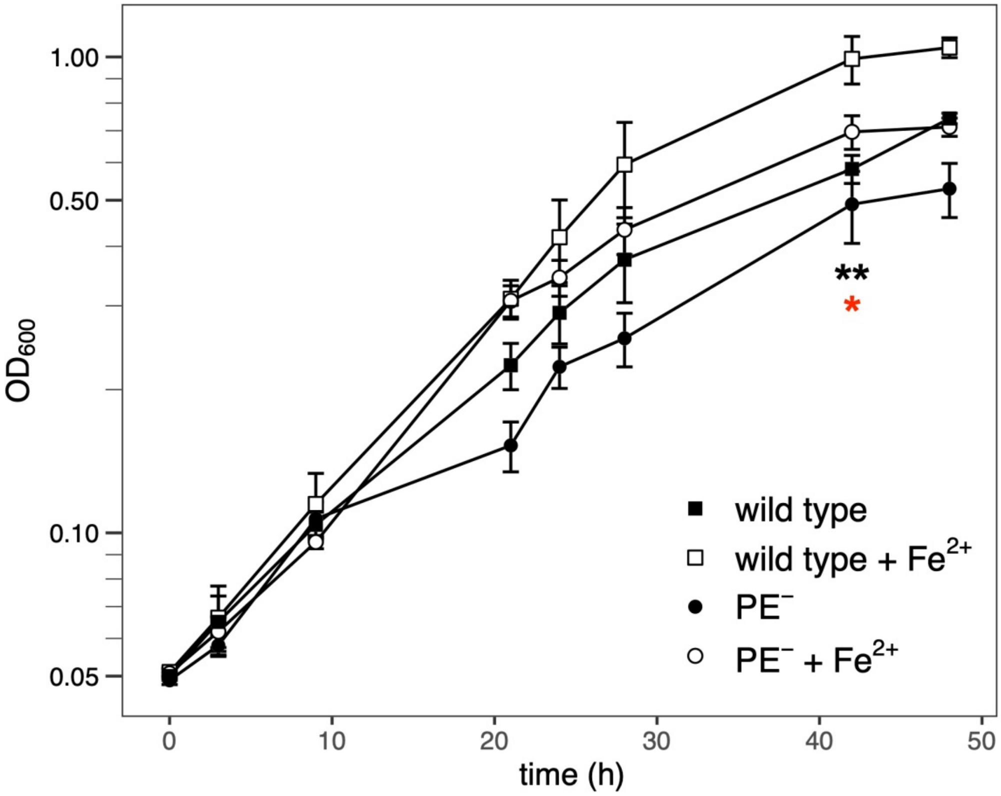

Fig. S1. Fe^{2+} contributes to rescue growth of PE-deficient mutant CS111 on minimal media. Third subcultivation of *S. meliloti* 1021 wild type (■,□) or PE-deficient mutant CS111 (●,○) on modified MOPS minimal medium containing $CaCl_2$ (5 mM), choline chloride (50 μM), in the absence (■.●) or presence of 1 μM $FeCl_2$ (□,○). Values represent means of three independent experiments and standard deviation is shown. Statistical analysis was performed by a two-way ANOVA with Tukey´s multiple comparisons test as described in Materials and Methods. Comparisons of wild type with wild type in the presence of $FeCl_2$ (black asterisks) and PE-deficient mutant with PE-deficient mutant in the presence of $FeCl_2$ (red asterisks) are shown for 42 h. Statistical significance is shown (* $p < 0.05$; ** $p < 0.01$).

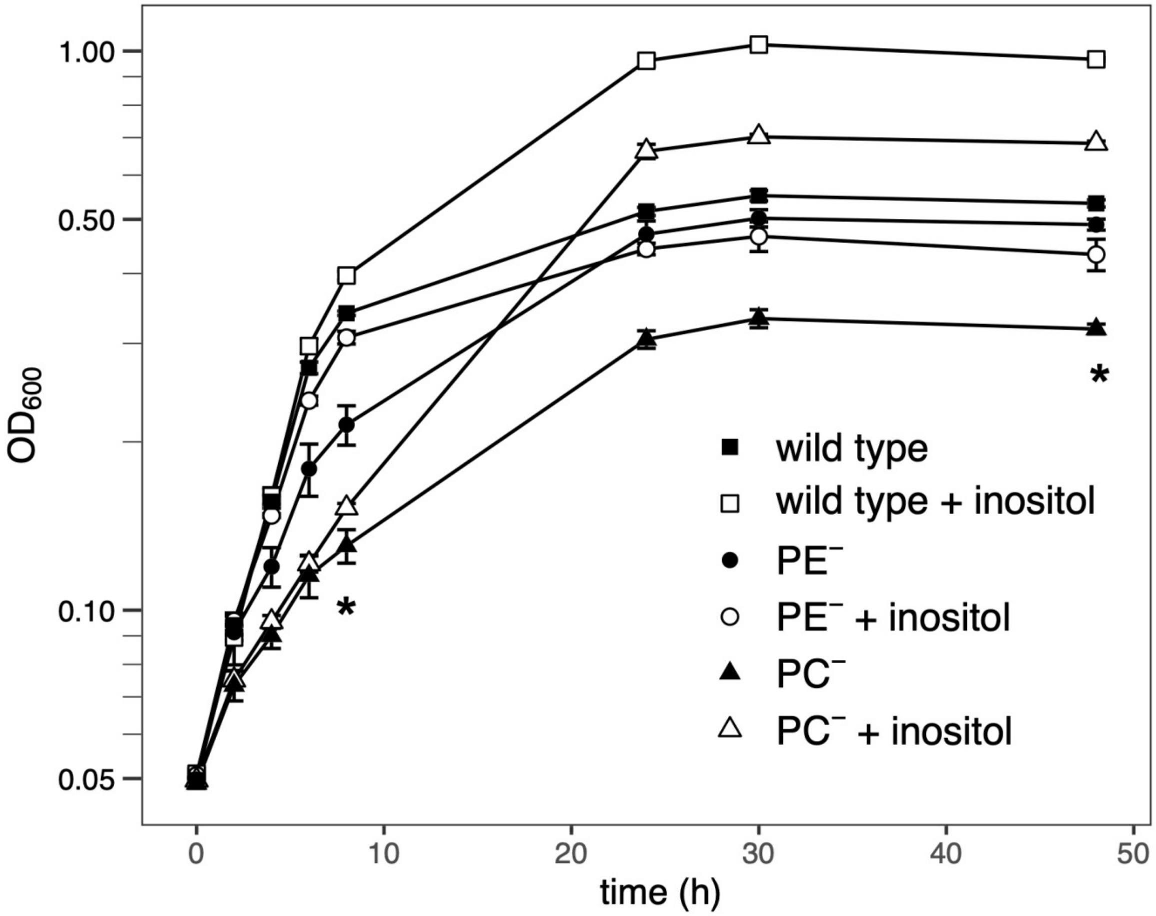

Fig. S2. *Myo*-inositol inhibits growth of PE-deficient mutant CS111. *S. meliloti* 1021 wild type (■,□), PE-deficient mutant CS111 (●,○), and the PC-deficient mutant OG10017 (▲,Δ) were cultivated on 1/20 LB/MC+ in the absence (■,●,▲) or presence of 4 mM *myo*-inositol (□,○,Δ). Values represent means of three independent experiments and standard deviation is shown. Comparisons of OD_{600} values at 8 or 48 h using ANOVA revealed significant differences between growth with and without inositol for each strain, with the highest p-value being 0.03. Statistical significance is shown (* $p < 0.05$).

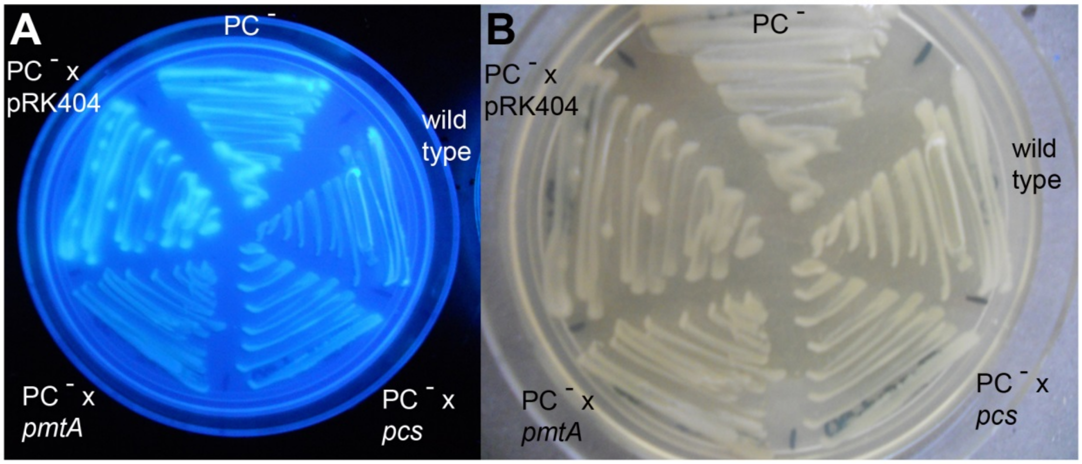

Fig. S3. Suppression of succinoglycan formation by PC-producing *S. meliloti* 1021 strains. Strains were cultivated for 72 h on LB/MC+-containing agar in the presence of Calcofluor and absence of tetracycline. *S. meliloti* 1021 (wild type), PC-deficient mutant OG10017, OG10017 complemented with *pmtA*-expressing plasmid pTB2042, OG10017 complemented with *pcs*-expressing plasmid pTB2532, and OG10017 harboring the empty broad host range plasmid pRK404. Upon UV excitation, only the PC-deficient strains showed increased fluorescence indicating overproduction of succinoglycan (A), whereas growth was similar for all five strains (B).

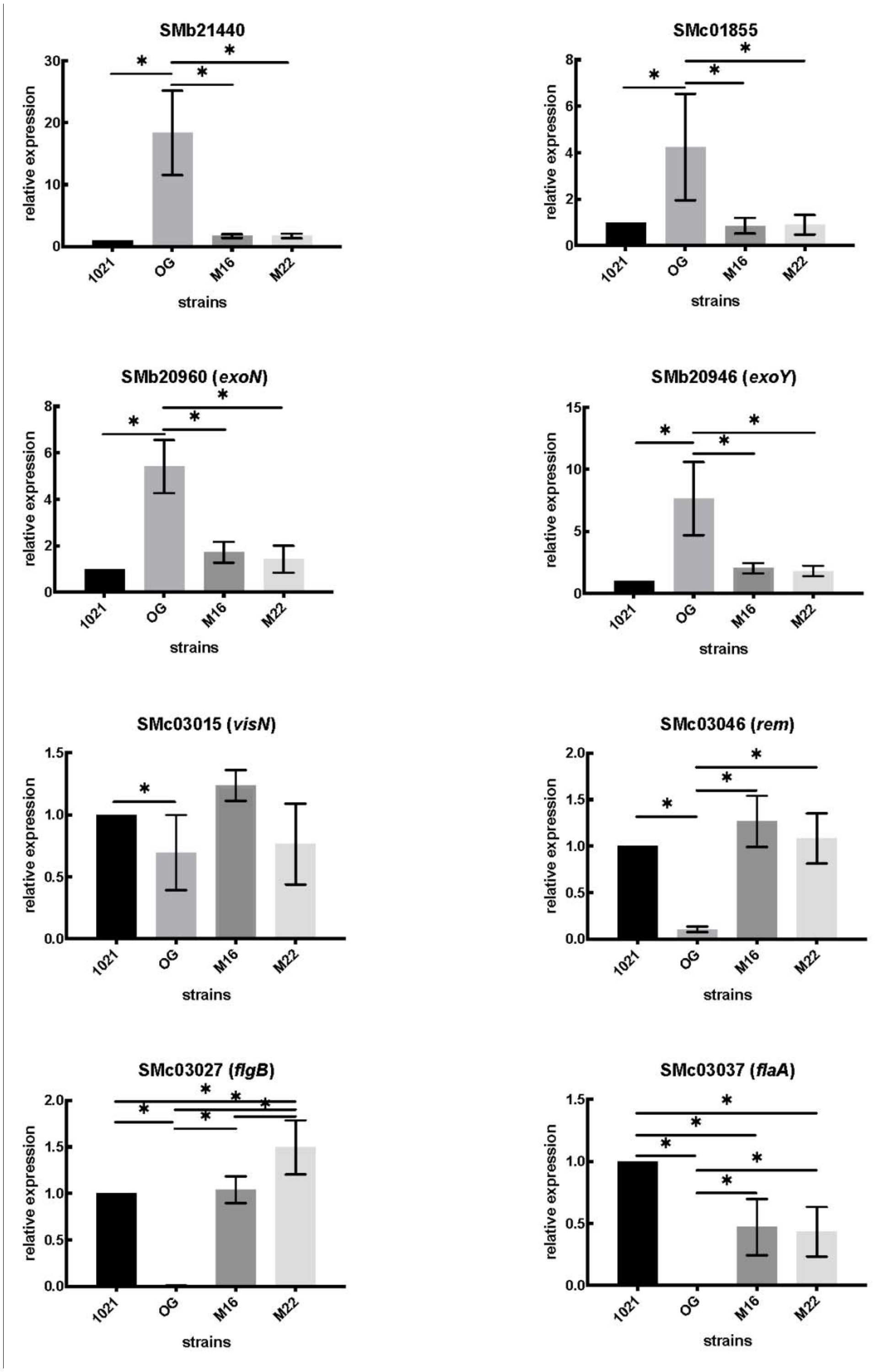

Fig. S4. Differential expression of selected *S. meliloti* genes in wild type, PC-deficient mutant OG10017, and correlated suppressor mutants M16 and M22 as determined by quantitative real-time PCR. The data represent averages of at least three independent experiments and standard deviation is shown. Data were analyzed by one-way ANOVA followed by Tukey test. Statistical significance is shown (* $p < 0.05$).

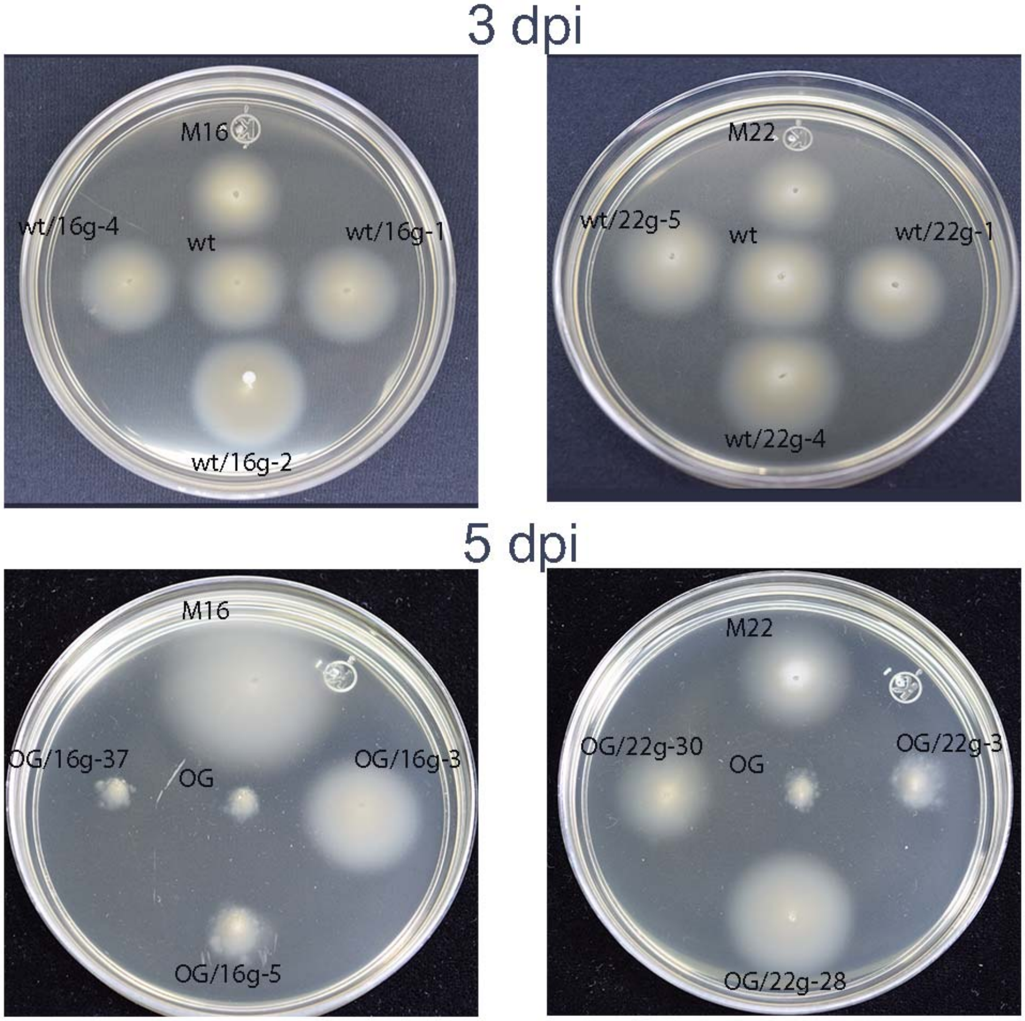

Fig. S5. Transduction of M16 and M22 versions of *exoS* increase swimming behavior in wild type and OG10017 backgrounds. Swimming behavior of wild type (wt), correlated suppressor mutants M16 and M22 and gentamicin-resistant transductants to the wild type (wt/16g or wt/22g) are shown 3 days post inoculation (3 dpi). Increased swimming behaviour of gentamicin-resistant wild type transductants is correlated with the cotransduction of the M16 (wt/16g-1, wt/16g-4) and M22 (wt/22g-4, wt/22g-5) versions of *exoS,* whereas swimming of gentamicin-resistant wild type transductants harbouring the wild type *exoS* version (wt/16g-2, wt/22g-1) is less (see also **Figure 9**). Also, Swimming behavior of OG10017 (OG), correlated suppressor mutants M16 and M22 and gentamicin-resistant transductants to OG10017 (OG/16g or OG/22g) are shown 5 days post inoculation (5 dpi). Increased swimming behaviour of gentamicin-resistant OG10017 transductants is correlated with the cotransduction of the M16 (OG/16g-3) and M22 (OG/22g-28, OG/22g-30) versions of *exoS,* whereas swimming of gentamicin-resistant wild type transductants harbouring the wild type *exoS* version (OG/16g-5, OG/16g-37, OG/22g-3) is less (see also **Figure 9**).

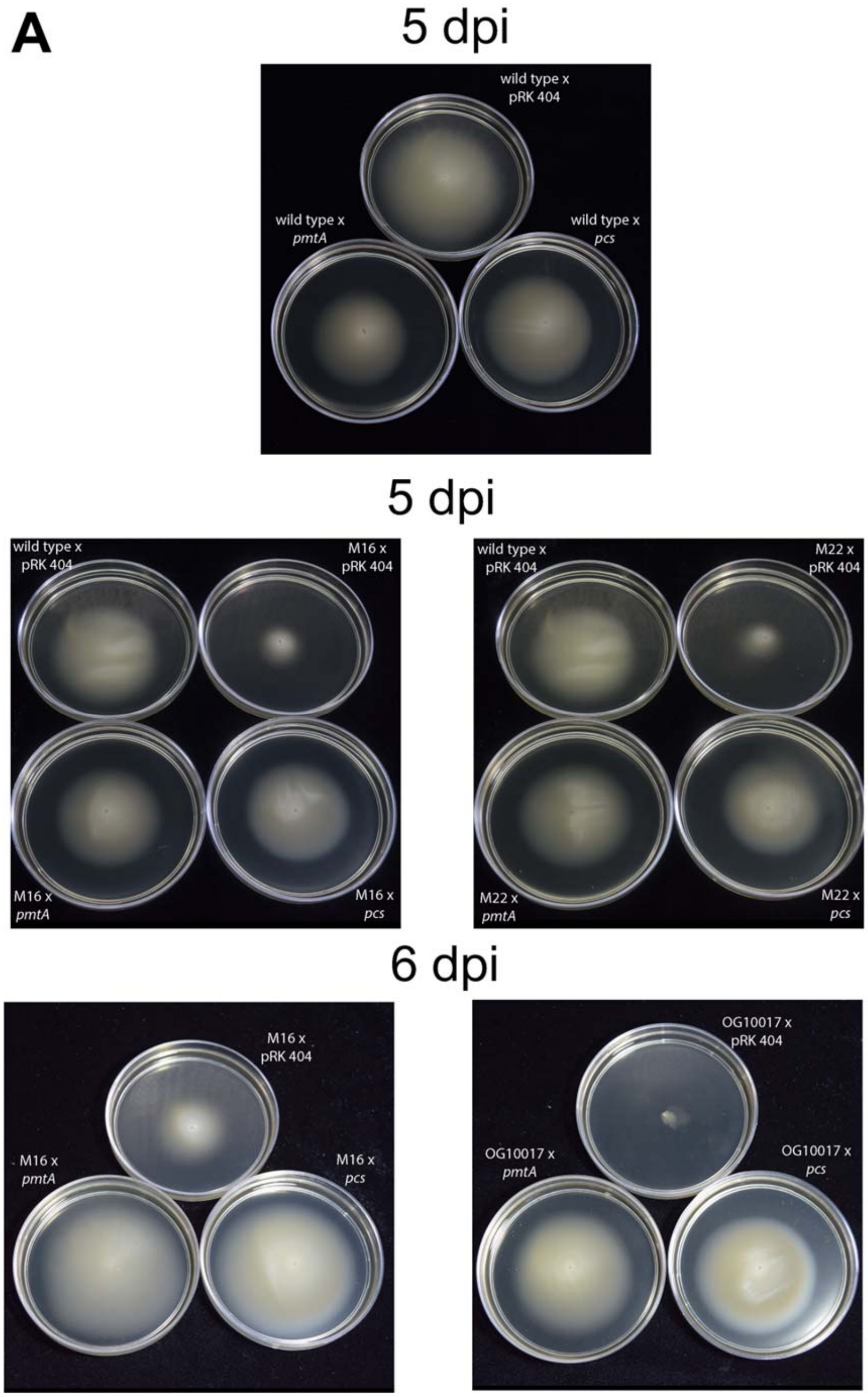
A
5 dpi
wild type x
pRK 404
wild type x
pmtA
wild type x
pcs
5 dpi
wild type x
pRK 404
M16 x
pRK 404
M16 x
pmtA
M16 x
pcs
wild type x
pRK 404
M22 x
pRK 404
M22 x
pmtA
M22 x
pcs
6 dpi
M16 x
pRK 404
M16 x
pmtA
M16 x
pcs
OG10017 x
pRK 404
OG10017 x
pmtA
OG10017 x
pcs

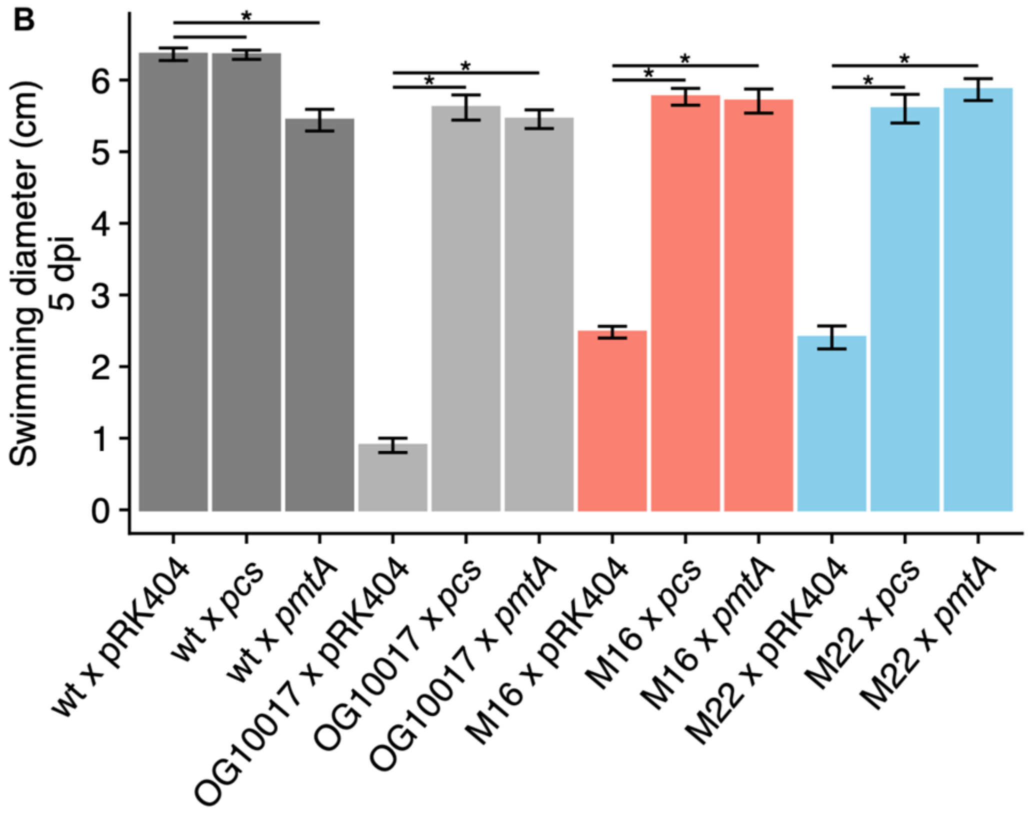

Fig. S6. Restoration of PC biosynthesis in correlated suppressor mutants M16 and M22 further increase swimming. (A) Swimming motility of *S. meliloti* 1021 strains was evaluated at 28˚C on LB/MC+-containing swim plates (0.3% agar) and analyzed after 5 or 6 days post inoculation (dpi) as indicated. *S. meliloti* 1021 (wild type; wt), PC-deficient mutant OG10017 (OG), correlated suppressor mutants M16 and M22 harbored the *pmtA*-expressing plasmid pTB2042 (*pmtA*), the *pcs*-expressing plasmid pTB2532 (*pcs*), or the empty broad host range plasmid pRK404 (pRK). (B) Quantification of swimming behavior. Colors indicate the *exoS* version of strains: wt (grey), M16 (red), M22 (blue). Values represent means of three independent experiments and standard deviation is shown. Pairwise comparisons were done using a Wilcox test and denoted by horizontal lines between the compared groups. Statistical significance is shown (* $p < 0.05$).

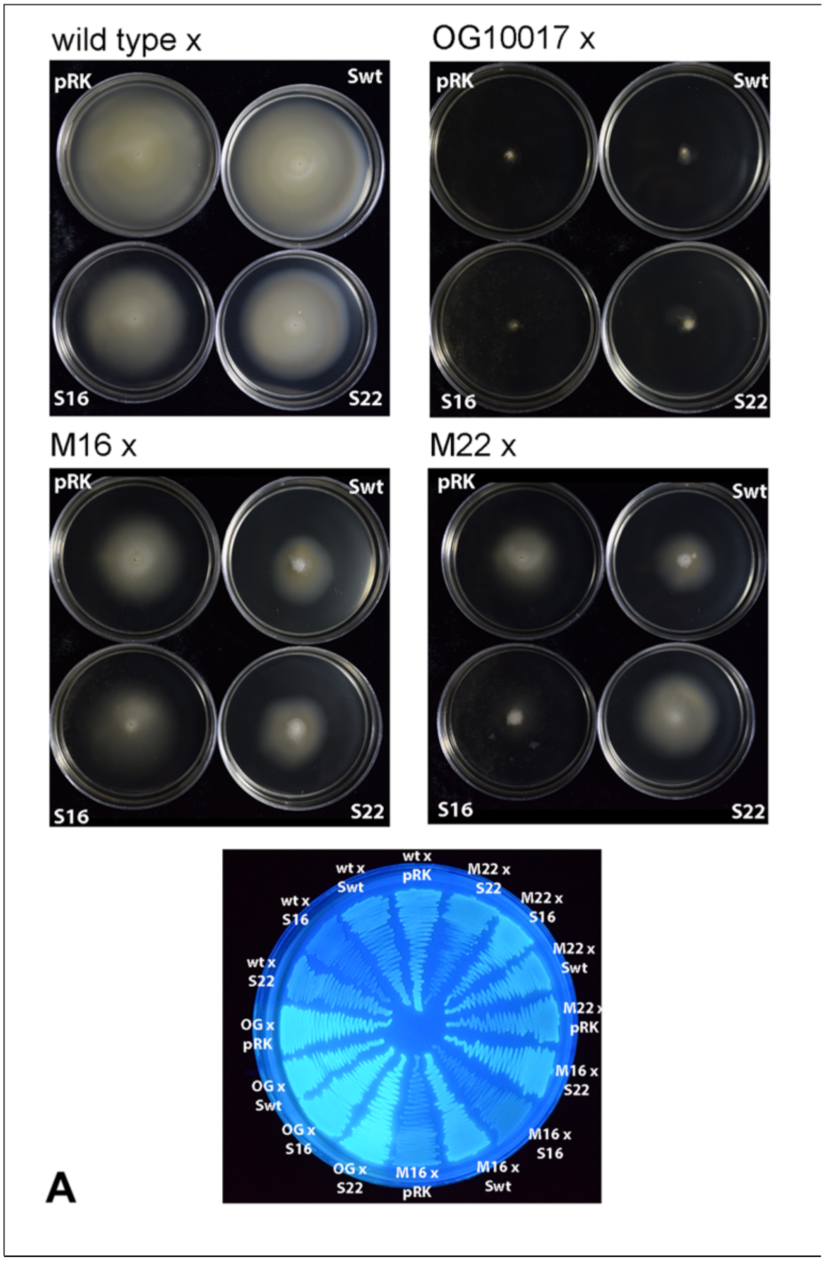
wild type x
pRK
Swt
S16
S22
OG10017 x
pRK
Swt
S16
S22
M16 x
pRK
Swt
S16
S22
M22 x
pRK
Swt
S16
S22
wt x pRK
wt x Swt
wt x S16
wt x S22
OG x pRK
OG x Swt
OG x S16
OG x S22
M16 x pRK
M16 x Swt
M16 x S16
M16 x S22
M22 x pRK
M22 x Swt
M22 x S16
M22 x S22
A

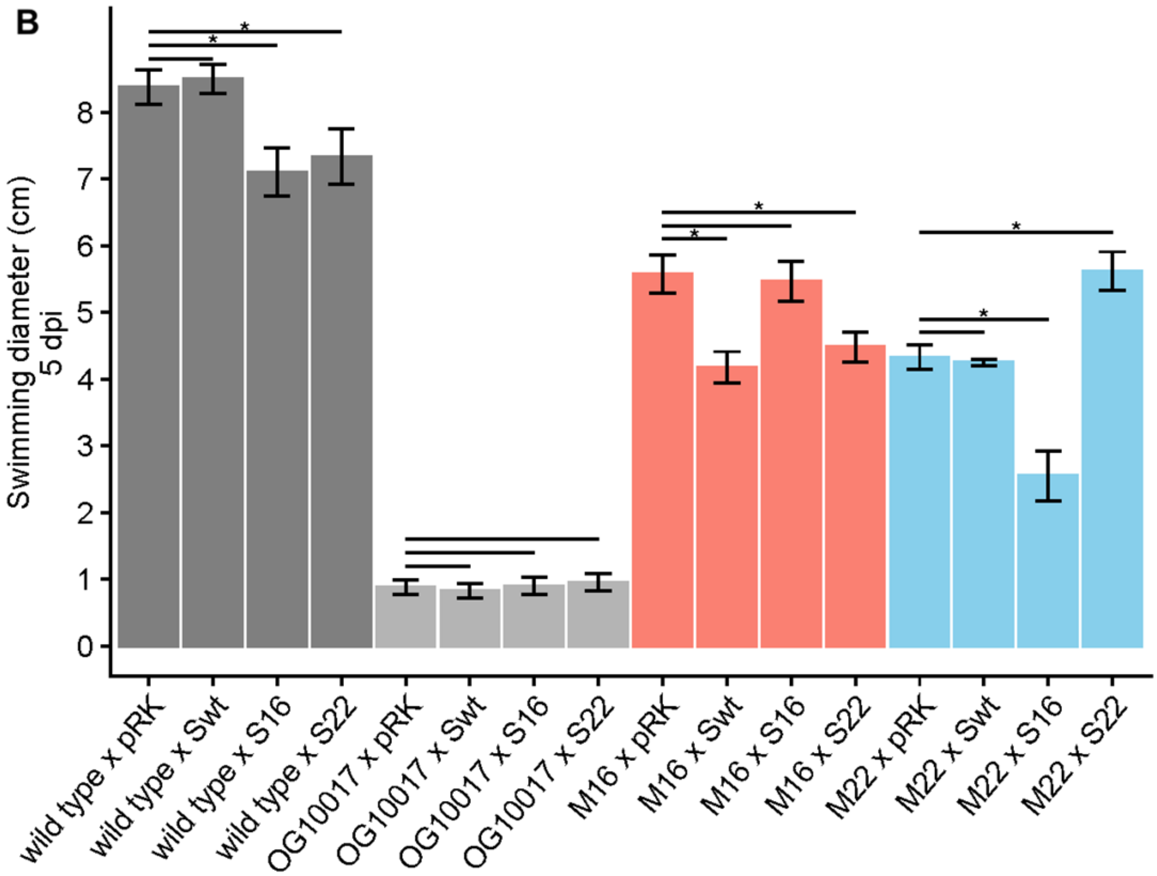

Fig. S7. Effect of different *trans*-expressed ExoS versions on succinoglycan formation and swimming in distinct *S. meliloti* strains. (A) Swimming motility of *S. meliloti* 1021 strains was evaluated at 28˚C on LB/MC+-containing swim plates (0.3% agar) and analyzed after 7 days post inoculation. *S. meliloti* 1021 (wild type; wt), PC-deficient mutant OG10017 (OG), correlated suppressor mutants M16 and M22 harbored the wild type *exoS*-expressing plasmid pJMV01 (Swt), the M16 type *exoS*-expressing plasmid pLMA16 (S16), the M22 type *exoS*-expressing plasmid pLMA22 (S22), or the empty broad host range plasmid pRK404 (pRK). For evaluation of succinoglycan formation, strains were cultivated for 72 h on LB/MC+-containing agar in the presence of Calcofluor, the absence of tetracycline, and fluorescence emitted upon UV excitation was visualized (bottom). (B) Quantification of swimming behavior. Colors indicate the *exoS* version of strains: wt (grey), M16 (red), M22 (blue). Values represent means of three independent experiments and standard deviation is shown. Pairwise comparisons were done using a Wilcox test and denoted by horizontal lines between the compared groups. Statistical significance is shown (* $p < 0.05$).
