# Supplementary material for: ExoS/ChvI Two-Component Signal-Transduction System Activated in the Absence of Bacterial Phosphatidylcholine
Source: Front Plant Sci. 2021 Jul 23;12:678976. doi: 10.3389/fpls.2021.678976 (PMC8343143; doi:10.3389/fpls.2021.678976)
Supplement: Supplementary file 2 [file Data_Sheet_2.PDF]

ExoS/ChvI two-component signal-transduction system activated in the absence of bacterial phosphatidylcholine

Supplementary Tables

Excel Table S1

Table S1. Complete list of *S. meliloti* genes differently expressed in the phosphatidylethanolamine-deficient mutant CS111 when compared to wild type. Tabular data (.xls) list of differentially expressed genes. M-values (log_2 ratio between both channels), P-values (*t* test) and A-values (log_2 of combined intensity of both channels) were also calculated with EMMA as described in Materials and Methods.

Excel Table S2

Table S2. Complete list of *S. meliloti* genes differently expressed in the phosphatidylcholine-deficient mutant OG10017 when compared to wild type. Tabular data (.xls) list of differentially expressed genes. M-values (log_2 ratio between both channels), P-values (*t* test) and A-values (log_2 of combined intensity of both channels) were also calculated with EMMA as described in Materials and Methods.

Table S3. Sequences of oligonucleotides used for quantitative real-time PCR.

Gene	Forward Primer (5´ to 3´)	Reverse Primer (5´ to 3´)
SMb21440	CTCGTTGCATCGCTGACC	TCGGGTTGGGTCTGTGTC
SMc01855	TTCTTCGCCGGGATTGGT	GCGTGGCAATGTCAGGTC
SMb20960 (*exoN*)	ATCCCAGTCCTTCCACGC	ACAGAACCATGTCGCGGA
SMb20946 (*exoY*)	CCGCAACGACGTCTCCTA	CGGCAGGGATCGTCTTGA
SMc03015 (*visN*)	TCCTTGATGCTGCTCTTC	CTCGGTCAGTTCGCATTC
SMc03046 (*rem*)	CGAAAGCCACATCAGCAAGC	ATTCCAGTCGATGCAGTAGCC
SMc03027 (*flgB*)	GAAAGCGTGCTTCAGAAC	CTGACTTCGGTCACATGC
SMc03037 (*flaA*)	CGATTATGTCAAGGTCCA	GCAATGGTGATGTCGATC
