# Supplementary material for: ExoS/ChvI Two-Component Signal-Transduction System Activated in the Absence of Bacterial Phosphatidylcholine
Source: Front Plant Sci. 2021 Jul 23;12:678976. doi: 10.3389/fpls.2021.678976 (PMC8343143; doi:10.3389/fpls.2021.678976)
Supplement: Supplementary file 3 [file Data_Sheet_3.PDF]

Table S4. Co-occurrence of the ExoR/ExoS/ChvI (RSI) invasion switch and bacterial PC biosynthesis genes within the Rhizobiales. As ExoR is encoded only in a subfraction of genomes that encode for ExoS or ChvI (Heavner et al., 2015), the presence of ExoR usually means that the complete RSI invasion switch is present in a given bacterium. In α-proteobacteria, PC can be synthesized by members of the rhodobacterial or sinorhizobial phospholipid *N*-methyltransferase (PmtA) families or members of the phosphatidylcholine synthase (Pcs) family (Geiger et al., 2013). Using ExoR of *S. meliloti* (ExoR; SMc02078), PmtA of *Rhodobacter sphaeroides* (rhodobacterial PmtA; L07247), PmtA of *S. meliloti* (sinorhizobial PmtA; SMc00414), or Pcs of *S. meliloti* (sinorhizobial Pcs; SMc00247), BLAST-P searches were performed on proteomes of selected α-proteobacteria. Candidates with alignment scores > 100 are listed with their assigned locus tags. Strains in blue lettering belong to the Rhizobiales whereas other α-proteobacteria are lettered in black.

Strain	ExoR	rhodobacterial PmtA	sinorhizobial PmtA	sinorhizobial Pcs
Magnetococcus marinus MC-1	-	-	-	-
Pelagibacter (SAR11 cluster)	-	-	-	-
Rickettsia bellii OSU_85_389	-	-	-	-
Rhodospirillum rubrum S1 ATCC11170	-	-	-	-
Magnetospirillum magnetotacticum MS-1	-	-	-	-
Gluconobacter oxydans 621H	-	GOX1862	-	-
Sphingomonas wittichii RW1	-	-	Swit_3372	-
Zymomonas mobilis ZM4	-	-	ZMO0776	-
Caulobacter vibroides CB15	-	-	-	-
Rhodobacter sphaeroides 2.4.1	-	L07247	-	DQL45_11410
Brucella abortus bv_1_9_941	BruAb1_0884	-	BruAb1_2102	BruAb2_0652
Bartonella tribocorum CIP_105476	BT_1529	-	-	BT_1173
Mesorhizobium loti MAFF303099	mll1100	-	mll4753	mll0506
Rhizobium sp_NGR234_ANU265	NGR_c13600	-	NGR_c36740	NGR_c14490
Sinorhizobium medicae WSM419	Smed_1153	-	Smed_1420	Smed_3542
Sinorhizobium meliloti 1021	SMc02078	-	SMc00414	SMc00247
Agrobacterium tumefaciens C58 Dupont	Atu1715	-	Atu0300	Atu1793
Rhizobium leguminosarum bv viciae_3841	RL2037	RL1338	RL0333	RL2370
Rhizobium leguminosarum bv trifolii WSM2304	Rleg2_1478	Rleg2_0843	Rleg2_4327	Rleg2_1714
Rhizobium etli CIAT_652	CH0001900	CH0001293	CH0000354	CH0002129

Rhizobium etli CFN_42	RHE_CH00733	RHE_CH01201	CH0000514<br>RHE_CH00316<br>RHE_CH00448	RHE_CH02079
Azorhizobium caulinodans ORS_571	AZC_2814	AZC_4349	AZC_0161	-
Nitrobacter hamburgensis X14	Nham_1754	Nham_0696	Nham_0157	Nham_2046
Bradyrhizobium japonicum USDA_110	AAV28_21095	bll6634	blr0681	AAV28_20155
Bradyrhizobium sp BTAi1	Bbta_4455	Bbta_1882<br>Bbta_6211	Bbta_0193	Bbta_4148
Bradyrhizobium sp ORS 278	BRADO4079	BRADO5893<br>BRADO5696	BRADO0162	BRADO3781
Rhodopseudomonas palustris BisB18	RPC_2495	RPC_0988<br>RPC_1444	RPC_0331	RPC_2618

References for Supplementary Table 4

Geiger, O., López-Lara, I.M., and Sohlenkamp, C. (2013). Phosphatidylcholine biosynthesis and function in bacteria. *Biochim. Biophys. Acta* 1831, 503-513. doi: 10.1016/j.bbalip.2012.08.009

Heavner, M.E., Qiu, W.-G., and Cheng, H.-P. (2015). Phylogenetic co-occurrence of ExoR, ExoS, and ChvI, components of the RSI bacterial invasion switch, suggests a key adaptive mechanism regulating the transition between free-living and host-invading phases in Rhizobiales. *PLoS ONE* 10 (8):e0135655. doi: 10.1371/journal.pone.0135655
